# LARS promotes osteosarcoma proliferation through leucine-dependent PRIM2 translation and DNA replication activation

**DOI:** 10.1186/s13046-026-03691-w

**Published:** 2026-03-14

**Authors:** Guannan Bai, Lin Zhang, Manli Zhao, Jingyao Zhang, Wenhao Chen

**Affiliations:** 1 Department of Orthopedic Surgery, Children’s Hospital, Zhejiang University School of Medicine, National Children’s Regional Medical Center, National Clinical Research Center for Child Health, 3333 Binsheng Road, Hangzhou, Zhejiang 310052 China; 2 The School of Public Health and Preventive Medicine, Monash University, 271 Collins Street, Melbourne, VIC 3205 Australia; 3 Department of Pathology, Children’s Hospital, Zhejiang University School of Medicine, National Children’s Regional Medical Center, National Clinical Research Center for Child Health, 3333 Binsheng Road, Hangzhou, Zhejiang 310052 China; 4 National Clinical Research Center for Child Health, 3333 Binsheng Road, Hangzhou, Zhejiang 310052 China

**Keywords:** Osteosarcoma, LARS, PRIM2, Metabolism reprogramming, Endoplasmic reticulum stress, Translation efficiency

## Abstract

**Background:**

Osteosarcoma (OS) is an aggressive bone malignancy in adolescents, with poor prognosis and limited survival improvement over decades, necessitating new therapeutic targets. Prior research identified Leucyl-tRNA synthetase (LARS) as critical for OS proliferation, prompting this investigation into its underlying mechanisms.

**Method:**

Utilizing clinical OS samples, we assessed LARS and primase p58 subunit 2 (PRIM2) expression via immunohistochemistry. *In vitro* studies employed OS cell lines for LARS/PRIM2 overexpression or knockdown, followed by functional assays: MTT, colony formation, EdU staining, Transwell migration/invasion, and flow cytometry for cell cycle/ROS/Ca^2^⁺ analysis. Xenograft models were used to evaluate tumor progression *in vivo*. Multi-omics analyses included transcriptome sequencing, proteomic profiling, and telomeric repeat amplification protocol-PCR to assess translational regulation. Stable isotope labeling by amino acids in cell culture (SILAC) determined leucine-dependent PRIM2 synthesis. Mechanisms were further probed using inhibitors and rescue experiments.

**Results:**

LARS expression is significantly elevated in OS, and its overexpression enhances proliferation but inhibits invasion and migration *in vitro* and *in vivo*. Conversely, LARS silencing in OS cells results in cell cycle arrest. Mechanistically, the upregulation of LARS in OS is associated with increased glycolysis and DNA replication and a reduction in endoplasmic reticulum stress, while elevating the oncogene PRIM2 through leucine-dependent translational control.

**Conclusion:**

Our findings highlight the crucial oncogenic role of the LARS/PRIM2 axis in promoting the pathogenesis of OS, primarily through the alleviation of endoplasmic reticulum stress and the activation of translation processes.

**Supplementary Information:**

The online version contains supplementary material available at 10.1186/s13046-026-03691-w.

## Introduction

Osteosarcoma (OS) is the predominant type of malignant bone tumor in children and adolescents [1]. This neoplasm is characterized by its propensity for rapid proliferation and early metastasis. Currently, the 5-year overall survival for patients with OS, following appropriate surgical resection and neoadjuvant chemotherapy, remains at approximately 50% [2]. The early onset of this tumor places substantial physical, psychological, and financial burdens on the families of affected children [3]. Although OS accounts for a relatively small proportion of solid malignant tumors in the pediatric population, it has garnered considerable attention due to its poor prognosis and the limited improvements in survival rates over recent decades [4].

Metabolic reprogramming is a hallmark of cancer, enabling the rapid growth and proliferation of malignant cells. Unlike normal cells, which metabolize glucose through oxidative phosphorylation under aerobic conditions, cancer cells predominantly generate energy via glycolysis even in the presence of adequate oxygen—a phenomenon known as the Warburg effect [5]. To meet the energy requirement, cancer cells also metabolize amino acids and fatty acids to produce adenosine triphosphate (ATP), highlighting the intricate relationship between amino acid metabolism and carbohydrate metabolism [6]. Recent studies have shown that amino acid metabolism plays a crucial role in OS progression through diverse mechanisms. High concentrations of glycine and D-serine in the tumor microenvironment (TME) suppress OS cell proliferation [7], whereas the mammalian target of rapamycin complex 1 (mTORC1) promotes their biosynthesis and concurrently inhibits apoptosis [8]. In contrast, reduced angiopoietin-like 4 (ANGPTL4) expression is associated with the intracellular accumulation of branched-chain amino acids and enhanced proliferation of OS cells [9]. Additionally, methionine has been implicated in promoting epithelial-mesenchymal transition (EMT) and malignant phenotypes of OS [10]. However, the development of novel metabolism-based therapeutic strategies for OS has plateaued. This stagnation may be attributed to the complex regulatory network governing metabolic reprogramming, which is further modulated by signals from the TME [11].

Aminoacyl-tRNA synthetase (ARS), extensively implicated in the regulation of cellular biology, possesses not only catalytic domains essential for protein synthesis but also binding domains for gene regulatory factors [12]. Abnormal expression of ARS has been observed in various tumors [13–16]. Leucyl-tRNA synthetase (LARS/LARS1), a member of the ARS family, has been shown to regulate mTORC1 activity and cell proliferation across various biological contexts. Specifically, LARS downregulation suppresses mTORC1 signaling and inhibits the proliferation of human embryonic cells [17]. In addition, silencing of LARS impairs leucine-dependent translation and signaling, thereby reducing tumor cell proliferation in non-small cell lung cancer and breast cancer models [18, 19]. Besides, our previous study revealed that LARS expression was significantly elevated in OS and negatively correlated with the survival rate of pediatric patients with OS. Moreover, silencing LARS markedly reduced the proliferation of OS cells [20]. However, the mechanism by which LARS facilitates the proliferation of OS cells has yet to be elucidated.

In this study, we demonstrate that LARS promotes the proliferation of OS cells through the augmentation of leucine-metabolism-associated glycolysis and the alleviation of endoplasmic reticulum (ER) stress. Additionally, we identify that the translation of the primase p58 subunit 2 (PRIM2), which is regulated by LARS, also contributes to the proliferation of OS cells. These insights may provide novel therapeutic strategies for pediatric patients with OS. The work has been reported in line with the TITAN criteria (the TITAN Guidelines 2025 – governing declaration, 10.70389/PJS.100082).

## Materials and methods

### Cell culture

The 143B (YDT-0722; RRID: CVCL_2270), SW1353 (YDT-0629; RRID: CVCL_0543), MG-63 (YDT-0400; RRID: CVCL_0426), U2OS (YDT-0678; RRID: CVCL_0042), HOS (YDT-0265; RRID: CVCL_0312), K7M2 (YDT-0317; RRID: CVCL_2130), and HEK-293 T (YDT-0019; RRID: CVCL_0063) for this study were sourced from INDIT Bio-Technology Co., Ltd. (Hangzhou, China). 143B cells were cultivated in 1640 medium (Gibco, USA, 12,633,020), and SW1353 cells were maintained in Leibovitz's L-15 medium (Gibco, USA, 11,415,064). U2OS cells were grown in McCoy's 5 A medium (Pricella, China, PM150710). MG-63, HOS, and HEK-293 T cells were cultured in minimum essential medium (MEM; BDBIO, China, C11095500BT), and K7M2 cells were maintained in DMEM medium (BDBIO, China, 02-5062EJ). All cell lines were cultured at 37 °C in a humidified atmosphere containing 5% CO₂. Culture medium was supplemented with 10% fetal bovine serum (FBS; SERANA, China, s-FBS-x-015) and 1% penicillin/streptomycin (Gibco, USA, 15,140–122).

### Concentrations and duration of reagent treatments

The mTOR inhibitor Rapamycin (MedChemExpress, USA, HY-10219) was dissolved in dimethyl sulfoxide (DMSO) at a ratio of 5 mg drug per 5.47 mL DMSO to prepare a 1 mM stock solution. It was applied to both 143B and SW1353 cells at a final concentration of 10 nM for 48 h. The reactive oxygen species (ROS) scavenger N-acetylcysteine (NAC; MedChemExpress, USA, HY-B0215) was dissolved in sterile phosphate-buffered saline (PBS) at a ratio of 20 mg drug per 12.25 mL PBS to generate a fresh stock solution and used at a final concentration of 10 mM for 1 h in 143B and SW1353 cells. Additionally, the PKR-like endoplasmic reticulum kinase (PERK) activator CCT020312 (Merck, Germany, 324,879) was dissolved in DMSO at a ratio of 1 mg drug per 1.54 mL DMSO to prepare a 1 mM stock solution, and was administered at final concentrations of 2 µM for 143B cells and 1 µM for SW1353 cells. For all experiments involving DMSO-dissolved reagents, an equivalent volume of DMSO was used as the vehicle control.

### Cell transfection

To establish transiently overexpressing OS cell lines for LARS, PRIM2, and ribosomal protein L23a (RPL23a), plasmids designed to overexpress LARS (NM_020117; RRID: Addgene_139690), PRIM2 (NM_000947; RRID: Addgene_74073), and plasmids overexpressing RPL23a (NM_000984; RRID: Addgene_134535), along with the empty pCDH vector (VT1480; RRID: Addgene_46969), were purchased from Youbio (Hunan, China). The human coding sequence (CDS) was synthesized, subjected to restriction enzyme digestion, and subsequently inserted into the pCDH vector to construct the overexpression plasmids. These plasmids were then transfected into OS cells using Lipofectamine 2000 (Invitrogen, USA, 11,668–019) and incubated for 24 h. For the generation of LARS or PRIM2 knockdown OS cell lines, siRNAs targeting LARS (siR-LARS), PRIM2 (siR-PRIM2), and a negative control siRNA (siR-NC) were purchased from Tsingke Biotech Co., Ltd. (Beijing, China). The above siRNAs were transfected into OS cells using Lipofectamine 2000 for 24 or 48 h. The sequences of these siRNAs are provided in Table 1.

**Table 1:** The sequences of siRNAs

Name	Sense	Antisense
siR-NC	UUCUCCGAACGAGUCACGUTT	ACGUGACUCGUUCGGAGAATT
siR-LARS1	GCUGUGCUUAUGGAGAAUAUA	UAUAUUCUCCAUAAGCACAGC
siR-LARS2	CCAGGGCUUUACCAAAGACAA	UUGUCUUUGGUAAAGCCCUGG
siR-LARS3	CCUCACUUUGACCCAAGCUAU	AUAGCUUGGGUCAAAGUGAGG
siR-PRIM2-1	CACGAAGAAGAGAUCAUAUUU	AAAUAUGAUCUCUUCUUCGUG
siR-PRIM2-2	CACGAAGAAGAGAUCAUAUUU	AAAUAUGAUCUCUUCUUCGUG
siR-PRIM2-3	GAAAUGGAUCUCCUUCGAUUU	AAAUCGAAGGAGAUCCAUUUC

To establish stable LARS- and RPL23A-overexpressing OS cell lines, the reconstructed lentiviral vector plasmids containing the target gene (specifically, Lenti-LARS containing the LARS gene and Lenti-RPL23a containing the RPL23a gene) and negative control plasmid (Lenti-control) were obtained from Youbio and sequence-verified. These plasmids were co-transfected with packaging plasmids into HEK-293 T cells to generate lentiviral particles. Additionally, the lentiviral vector plasmid containing the target gene sh-LARS (Lenti-sh-LARS) or negative control (Lenti-sh-control) was used to construct a stable LARS knockdown OS model. Co-transfection was performed using Lipofectamine 2000 and incubated for 48 h. After transfection, the viral supernatant was collected, filtered, and transduced to OS cells at 60%−70% confluency with Lipofectamine 2000 for 24 h. Following transfection, the medium was refreshed, and puromycin (2.5 µg/mL, Yuanye, China, S17055) was applied for 7–10 days to select positive cells.

### Methyl Thiazolyl Tetrazolium (MTT) assay

The proliferation of OS cells was assessed using the MTT assay. OS cells were cultured in 96-well plates at a concentration of 1 × 10^4^ cells per well and incubated for 24 h. To assess the cytotoxicity of CCT020312 in OS cells (143B and SW1353), cells were seeded in 96-well plates at a density of 1 × 10^4^ cells/well (100 μL/well) and incubated overnight, followed by treatment with CCT020312 at concentrations ranging from 0 to 5 μM for 24 h. Subsequently, a 0.5 mg/mL MTT solution (Sigma, USA, M2128) was added to the cells, followed by an additional incubation period of 3 h. To dissolve the resulting formazan crystals, 150 μL of DMSO (Sigma, USA, D2650) was added to each well, and the plates were agitated for 10 min. Cell viability was evaluated by measuring the optical density (OD) at 570 nm with a microplate reader (Thermo Fisher, USA, 51,119,570).

### Colony formation assay

OS cells were plated in 6-well plates at a density of 5 × 10^3^ cells per well. To facilitate colony formation, the cells were kept in a specified environment (5% CO₂, 37 °C) for 12 days, with the medium being changed every 3 days. In succession, the forming colonies were fixed and stained using methanol and 0.5% crystal violet (RRID: SCR_010476; Aladdin China, C110703). They were then observed under a microscope (RRID: SCR_025025; Olympus, Japan, CKX53).

### Flow cytometry assay

OS cells were harvested during the logarithmic growth phase after gene overexpression or knockdown interventions, and cell cycle analysis was performed immediately at the indicated time points following these treatments. Cells were then collected by centrifugation and washed with PBS. Cell cycle analysis was performed using a cell cycle detection kit (Beyotime, China, C1052-50 T) according to the manufacturer's instructions. Briefly, the cells were fixed in 70% ethanol overnight, then stained with propyl iodide (PI) stain solution (dye buffer: 0.5 mL/sample, PI: 25 μL/sample, and RNase A: 10 μL/sample) to incubate at 37 °C for 30 min away from light before flow cytometry analysis (RRID: SCR_025067; Beckman Coulter, USA, CytoFLEX) with a 488 nm laser.

To measure the Ca^2+^ level in the ER, OS cells were trypsinized, stained with Mag-Fluo-AM (GENMED, China, GMS10267.1–20 T), and incubated at 37 °C. Following centrifugation, the pellet was resuspended in PBS and then subjected to flow cytometric analysis, detecting green fluorescence at 490 nm excitation and 525 nm emission.

For ROS detection, OS cells were collected by centrifuging in serum-free MEM, stained with a 1:2000 diluted ROS probe (Beyotime, China, S0033S), and then incubated at 37 °C for 30 min. After being washed and resuspended twice with PBS, the cells were filtered, resuspended in a serum-free medium, and loaded into flow cytometry tubes for ROS quantification.

### Transwell assay

The migration and invasion capabilities of OS cells were evaluated using Transwell assays. For the migration assays, OS cells were seeded at 1 × 10^4^ cells per well in the upper Transwell chamber (RRID: SCR_008983; Millipore, USA, CLS3396) without serum. The bottom chamber was filled with a nutrient medium consisting of 10% FBS. After 24 h of incubation, the cells were immobilized using methanol and stained with crystal violet dye. Invasion assays were performed by adding Matrigel (Corning, USA, 354,234) to the upper chamber half an hour in advance. The rest of the procedure was the same as the migration experiment. Observation and imaging of cell migration and invasion were conducted using a microscope (Olympus Corp, China, IX73, magnification: 100 ×).

### 5-Ethynyl-2'-deoxyuridine (EdU) staining assay

The Cell-Light EdU Apollo643 *In Vitro* Kit (RIBBIO, China, C10310-2) was used for the EdU staining assay. OS cells were plated in 24-well dishes at a concentration of 5 × 10^4^ cells per well, washed with PBS, and then incubated in a serum-deprived medium supplemented with 10 μmol/L EdU at 37 °C for 2 h. The cells were then fixed with 4% paraformaldehyde at 4 °C for 15 min. Subsequently, they were stained with Apollo solution and DNA staining solution at room temperature. The fluorescence intensity of EdU staining was quantitatively assessed using Integrated Optical Density (IOD) analysis. EdU-labeled images of cells from each group were captured using a fluorescence microscope (Nikonbm, Japan, SMZ18, magnification: 630 ×). Regions of interest (ROIs) corresponding to the cell nuclei were selected for analysis, and the integrated optical density of fluorescence intensity was calculated using ImageJ software.

### Immunohistochemistry (IHC) assay

A total of 14 OS clinical samples were collected from Children’s Hospital, Zhejiang University School of Medicine, including 8 paired peritumoral and primary tumor samples and 6 metastatic tumor samples derived from patients with synchronous lung metastases. The clinicopathological characteristics of these OS patients included ages ranging from 8 to 18 years, with locally advanced disease classified as Enneking stage IIB or stage III. Detailed clinical information is summarized in Table S1. Ethical approval of clinical samples for this study was provided by the Ethical Committee of Children's Hospital, Zhejiang University School of Medicine.

IHC assays were used to analyze the expression of the target protein in clinical sample tissues and xenograft tissues. Tissue samples were preserved in 4% paraformaldehyde for one week, dehydrated using ethanol and xylene, and then embedded in paraffin. The paraffin-embedded 5 μm sections underwent deparaffinization and rehydration and were then subjected to antigen retrieval. After natural cooling, the slides were placed in PBS (pH 7.4) and washed on a decolorizing shaker three times for 5 min each. Primary antibodies were incubated overnight, followed by secondary antibody incubation. The staining and re-staining were completed with 3,3'-Diaminobenzidine (DAB; Servicebio, China, G1212) and hematoxylin (Servicebio, China, G1004). The expression of the target protein was visualized under a microscope (Olympus Corp, Japan, CX31, magnification: 800 × or 400 ×) and compared with the control group. The results were quantitatively evaluated using IOD analysis. The antibodies used in this experiment are presented in Table 2.

**Table 2:** Details of antibodies used for IHC

Name	Brand	Number
LARS	proteintech	21,146–1-AP
CLUT4	proteintech	81,463–1-RR
HK2	CST	2867 T
PRIM2	proteintech	11,788–1-AP

### Western Blotting (WB) analysis

Proteins were extracted from pretreated cells on ice using RIPA lysis buffer, and their total concentration was determined using the BCA protein assay kit (Beyotime, China, P0012). Protein samples, each containing 20 μg, underwent separation via sodium dodecyl sulfate–polyacrylamide gel electrophoresis (SDS-PAGE) and were subsequently transferred onto a polyvinylidene difluoride (PVDF) membrane (RRID: SCR_008983; Millipore, GRE, IPVH00010). After the blocking procedure to hinder non-specific interactions and the incubation phase for specific antibody binding, involving both primary and secondary antibodies, the protein bands were observed using the ECL kit (Biosharp, China, BL520B). The antibodies used in this experiment are presented in Table 3.

**Table 3:** Details of antibodies used for WB

Name	Brand	Number
LARS(RRID: AB_10733878)	Proteintech	21,146–1-AP
CLUT4(RRID: AB_2882186)	Proteintech	81,463–1-RR
HK2(RRID: AB_2882294)	CST	2867 T
PRIM2(RRID: AB_2169380)	Proteintech	11,788–1-AP
β-actin(RRID: AB_2750915)	CST	4970
PERK(RRID: AB_2770854)	ABclonal	A21255
p-PERK(RRID: AB_2576881)	Thermo	MA5-15,033
p-mTOR(RRID: AB_10888105)	ABCAM	ab109268
mTOR(RRID: AB_2882219)	proteintech	66,888–1-Ig
ATF4(RRID: AB_2058598)	Proteintech	60,035–1-lg
CHOP(RRID: AB_2292610)	Proteintech	15,204–1-AP
Ki-67(RRID: AB_2861195)	CST	9449S
PCNA(RRID: AB_2883829)	Proteintech	60,097–1-Ig
MCM2(RRID: AB_10859977)	Abcam	AB108935
ORC1(RRID: AB_628052)	Santa Cruz	sc-23887
Anti-mouse IgG, HRP—linked Antibody (RRID: AB_330924)	CST	7076P2
Anti-rabbit IgG, HRP—linked Antibody(RRID: AB_2099233)	CST	7074P2

### Real-time quantitative Polymerase Chain Reaction (RT-qPCR)

RNA extraction was performed using Trizol reagent (RRID: SCR_008406; Ambion, China, 15,596,018). This involved phase separation with chloroform and precipitation of RNA using isopropanol (RRID: SCR_008452; Acros Organics, USA, 327,270,010). Subsequently, the RNA precipitate was washed with 75% ethanol, dried, and dissolved in RNase-free water. Following this, the RNA concentration was assessed. The synthesis of complementary DNA (cDNA) was facilitated using the PrimeScript™ RT reagent Kit (RRID: SCR_021372); TAKARA, Japan, RR037Q). Then, RT-qPCR was executed utilizing the TB Green™ Premix Ex Taq™ II kit (RRID: SCR_021372; TAKARA, Japan, RR820S), with β-actin as the reference gene. The relative expression levels were determined employing the 2^⁻ΔΔCT^ method. The primer sequences utilized in this study are provided in Table 4.

**Table 4:** The sequences of RNA primers

Names	Forward primer	Reverse primer
β-actin	AGCAGTTGTAGCTACCCGCCCA	GGCGGGCACGTTGAAGGTCT
PRIM2	TACCAAATCCTTCCCACC	GTTAAACCAATGCCCTTC
LARS	AATGGCGTGGTGCCTGTTGT	TCTTCAAGTCTCTGAGGGCA

### Determination of glucose, lactic acid, and leucine levels

The cell suspension underwent centrifugation at 1000 rpm for 10 min, and the supernatant was discarded. The pellet obtained was washed twice with PBS, followed by another centrifugation step at 1000 rpm for 10 min. Subsequently, the cells were resuspended in PBS and lysed utilizing an ultrasonic cell disruptor (JIERUIAN, China, JRA-150X). The resultant supernatant was then collected for subsequent analysis. The glucose content was measured using a glucose assay kit (Solarbio, China, BC2505), following the manufacturer's guidelines. The lactic acid content was assessed with a lactic acid test kit (Solarbio, China, 2235), and the free leucine levels in cell supernatants were detected using a leucine detection kit (RRID: SCR_012931; Abcam, UK, ab234627), following the respective manufacturer's instructions.

### Construction of the xenograft tumor models* in vivo*

Ethical approval of animal experiments for this study was provided by the Ethical Committee of Zhejiang University Animal Experimental Center. A total of 36 female nude mice (5 weeks old) were purchased from Vital River Laboratory Animal Technology Co., Ltd. (RRID: SCR_003792; Beijing, China) and housed under specific pathogen-free conditions with a 12 h light/dark cycle at 22 °C for one week before experimentation.

For subcutaneous xenograft models, 143B cells stably overexpressing LARS (Lenti-LARS) or control cells (Lenti-control) were subcutaneously injected into nude mice at a density of 5 × 10⁶ cells per mouse in 200 μL PBS. Similarly, MG-63 cells with stable LARS knockdown (Lenti-sh-LARS) or corresponding control cells (Lenti-sh-control) were injected subcutaneously using the same cell number and volume to establish LARS-silenced xenograft models. For the lung metastasis model, K7M2 cells stably overexpressing LARS (Lenti-LARS) or control cells (Lenti-control) were injected via the tail vein at a density of 5 × 10^5^ cells per mouse in 200 μL PBS. Tumor growth was monitored, and tumor volume was calculated using the formula: 0.5 × length (mm) × width^2^ (mm^2^). After 5 weeks, mice were euthanized, and tumors were excised for further analysis.

For the lung metastasis model, lungs were harvested from euthanized mice, fixed in 4% paraformaldehyde, and fully spread with all five lobes (left 1 lobe, right 4 lobes) flattened without folding to avoid obscuring lesions; metastatic nodules were counted by stereomicroscopy (Leica M80, 40 ×) as discrete, round or ovoid, white/off-white, firm nodules with clear margins from normal pink lung tissue, with fused lesions counted as a single focus and total nodules summed from both lungs. All counts were independently performed by two blinded investigators and averaged to quantify the final metastatic burden.

### Proteomic profiling

SW1353 cells with stable overexpression of LARS, and their control cells, were analyzed following cell lysis using a buffer consisting of 8 M urea and 1% protease inhibitors. Following lysis, the samples underwent sonication and were centrifuged at 15,000 g for 10 min at 4 °C. The concentration of proteins was determined through a BCA assay kit. Equivalent protein quantities were precipitated with 20% trichloroacetic acid (TCA; RRID: SCR_012449; Aladdin, China, A291704) at 4 °C for 2 h, followed by centrifugation and acetone washing. Then, samples were resuspended in TEAB buffer (RRID: SCR_001287; MERCK, China, T7408), digested overnight with trypsin at 37 °C, and reduced with 5 mM DTT for 30 min. Iodoacetamide (IAA; MERCK, China, I6125) was added at a concentration of 15 mM for a light-protected reaction. The obtained samples were subjected to proteomic sequencing analysis at Hangzhou COSMOSWISDOM Technology Co., Ltd.

### Transcriptome sequencing

SW1535 cells that stably overexpressed LARS were harvested, and the total RNA was isolated using the Trizol reagent. The RNA's concentration, purity, and integrity were assessed using a Nucleic Acid Protein Detector (RRID: SCR_025369; NanoDrop, USA, ND-1000) and a Cell Analyzer (RRID: SCR_010917; Agilent, USA, Bioanalyzer 2100). mRNA was purified using oligo (dT) magnetic beads (Dynabeads Oligo; Thermo Fisher, USA, 25–61,005) and subsequently fragmented. cDNA was synthesized using a reverse transcriptase (RRID: SCR_008410; Invitrogen, USA, 1,896,649) and amplified by PCR to construct a library. Finally, the obtained samples were subjected to transcriptome sequencing at Hangzhou LC-Bio Technology Co., Ltd.

### Sucrose density gradient centrifugation assay

The OS cells were gathered, rinsed three times with ice-cold PBS, and scraped off the culture dish. After centrifugation at 5000 rpm for 3 min at 4 °C and discarding the supernatant, 500 µl of lysis buffer (comprising 50 mM Tris, 300 mM NaCl, 30 mM EDTA, and 0.5% Triton X-100) was introduced. The mixture was then incubated on ice for 10 min. The lysate was mixed and transferred to gradient centrifuge tubes containing sucrose solutions (10%, 20%, 30%, and 45%), adjusted to a final volume of 30 mL with polysome buffer (130 mM KCl, 50 mM Tris, 10 mM MgCl_2_). Centrifugation was performed at 2000 g for 10 min at 4 °C. The supernatant was gathered and divided equally among 20 tubes. The absorbance of the mRNA at 260 nm was then measured using a spectrophotometer (Thermo Scientific, USA, ND-NDL-2YRW-CCC).

### Telomeric repeat amplification protocol-PCR (TRAP-PCR) assay

Ribosome-bound mRNA complexes were isolated from RPL23a-overexpressing 143B cells that had been transfected with either LARS overexpression plasmid or siR-LARS for 24 h using GFP-Trap Magnetic Particles (RRID: SCR_008986; Proteintech, China, gtdk-20). Cells were washed twice with ice-cold PBS containing 100 μg/mL cycloheximide (CHX) to arrest ribosomes on mRNA. For cytoplasmic ribosome isolation, cells were lysed in 200 μL ice-cold lysis buffer supplemented with 1 × protease inhibitor cocktail and 1 mM phenylmethanesulfonyl fluoride (PMSF). For the purification process, follow the manufacturer's instructions. A segment of 500 ng from the total lysate derived from the transfected cells underwent incubation with 25 μL of magnetic bead suspension at 4 °C for 1 h, with continuous end-over-end rotation. The beads were subsequently washed 3 to 5 times with ice-cold wash buffer (10 mM Tris/Cl, pH 7.5, 150 mM NaCl, 0.05% Nonidet P-40 Substitute, 0.5 mM EDTA). Bound proteins were eluted with 2 × LDS lysis buffer under boiling conditions.

For normalization, input samples (10% of the total lysate) and IgG immunoprecipitation controls were included in each experiment. Relative enrichment was calculated by normalizing to the input and subtracting IgG background signals. mRNA products were then purified from the ribosome-mRNA complexes using an RNA purification kit (RRID: SCR_021372; Takara, Japan, 9108Q). The abundance of PRIM2 mRNA was quantified via RT-qPCR, and the data were normalized against the spike-in controls and the negative control samples.

### Stable isotope labeling by amino acids in cell culture (SILAC)

SILAC experiments were performed to explore the leucine-dependent translational regulation of PRIM2 by LARS. SW1353 cells transfected to overexpress LARS or control vectors were cultured in SILAC medium supplemented with either heavy stable isotope-labeled L-leucine (L-Leucine-^13^C₆) or natural L-leucine. After 6 passaging culture periods to allow for protein labeling and expression modulation, cells were harvested. Proteins were extracted, and tryptic digestion was carried out. Mass spectrometric analysis was performed on a Q Exactive HF-X system (Thermo Fisher, USA) with MaxQuant (version 2.0.3.0) for peptide identification. PRIM2 synthesis rates were quantified by heavy/light (H/L) ratio comparison. The SILAC experiment was performed by Yida Precision (Hangzhou) Technology Co., Ltd.

### Bioinformatic analyses

#### Single-cell RNA sequencing (scRNA-seq) analysis

The scRNA-seq data of OS were obtained from the Gene Expression Omnibus (GEO) database under accession number GSE152048, which contains 9 samples, including 7 primary and 2 lung metastatic lesions. Raw gene expression matrices from each sample were imported into "R" and processed using the "Seurat" package (version 5.3.0). Cells expressing fewer than 200 genes or exhibiting mitochondrial gene expression exceeding 25% of total counts were excluded to remove low-quality cells. The expression data were normalized using the NormalizeData function, and 2,000 highly variable genes were identified for downstream analysis. Principal component analysis (PCA) was performed using default Seurat parameters. To minimize batch effects arising from multiple samples, the Harmony algorithm was applied to the PCA embeddings for correction. Based on the corrected principal components, a shared nearest neighbor (SNN) graph was constructed, followed by clustering analysis. Two-dimensional visualization of the cell distribution was performed using Uniform Manifold Approximation and Projection (UMAP). Cell type annotation was carried out according to the expression profiles of canonical marker genes. For osteoblastic cell subpopulations, copy number variation (CNV) analysis was performed using the "inferCNV" package (version 1.18.1). Based on CNV scores, osteoblastic cells were classified into malignant and normal groups. Ultimately, eleven major cell clusters were identified, and UMAP projections were generated to illustrate the global transcriptional landscape and cluster distribution. To compare the cellular composition between primary and metastatic lesions, we downloaded relevant samples from two GEO datasets: primary lesions included BC2, BC3, BC5, BC6, BC16, BC21, and BC22, which were obtained from GSE152048; metastatic lesions included BC10 and BC17 (from GSE152048) as well as S0058, S0059, S0217, and S0218 (from GSE270231); the proportion of each cell type was calculated and visualized using stacked bar plots generated by the "ggplot2" package (version 4.0.0).

To investigate the potential role of LARS in OS progression, its expression pattern was analyzed in malignant osteoblastic cells, and differential expression between primary and metastatic lesions was assessed using the Wilcoxon rank-sum test, with spatial distribution visualized via "FeaturePlot" in Seurat. The AUCell package was employed to assess the activity of key biological processes, including cell proliferation potential, ER stress response, and glycolytic metabolism, across different cell populations. For subgroup analyses, malignant osteoblastic cells from primary lesions were stratified into LARS high and low groups based on median LARS expression, and AUCell scoring was used to assess differences in these biological processes between the two groups. Differential gene expression analysis between primary and metastatic malignant osteoblastic cells was conducted using the DESeq2 (RRID: SCR_012929) package (version 1.48.1). Genes with an adjusted *p*-value (*p*_adj) < 0.05 and an absolute log₂foldchange (|log₂FC|) > 1 were considered significantly differentially expressed. Functional enrichment analysis of these differentially expressed genes (DEGs) was performed using Gene Ontology (GO) (RRID: SCR_002811) annotation and Kyoto Encyclopedia of Genes and Genomes (KEGG) (RRID: SCR_012773) pathway analysis. Enrichment results were visualized using bar plots and bubble plots, while volcano plots were generated to depict the global distribution of DEGs.

#### GEO and TCGA data analysis

Gene chip data for OS were downloaded from the GEO database. Survival analysis of LARS in sarcoma (*n* = 259) was performed using the Kaplan–Meier Plotter database (https://kmplot.com/analysis/), with overall survival as the endpoint and the optimal cutoff value automatically determined. OS RNA-seq data were downloaded from the Cancer Genome Atlas (TCGA) (RRID:SCR_003193) database (https://portal.gdc.cancer.gov/). Tumor samples were categorized into high and low LARS expression groups based on the median expression value of LARS in OS samples. DEGs were identified using the "DESeq2" package in R. DEGs between tumor samples with high and low LARS expression were analyzed with thresholds set at *p* < 0.05 and |log_2_FC|> 1.5. Additionally, transcriptome sequencing was performed on SW1353 cells stably overexpressing LARS and their corresponding controls to identify DEGs. Differentially expressed proteins (DEPs) were characterized through proteomic analysis of SW1353 cells overexpressing LARS and their controls using the same statistical criteria. Heatmaps of DEGs or DEPs were generated using the "pheatmap" R package, and volcano plots depicting upregulated and downregulated genes or proteins were created with "ggplot2". Additionally, the proportion of leucine residues in the top 15 DEPs identified from proteomic analysis was calculated using the Expasy website (https://www.expasy.org/). The absorption peaks for both the Lenti-LARS and Lenti-control groups were visualized using the "ggplot2" package in R. A dot plot was created to show the overlap between DEGs from Sucrose density gradient centrifugation and DEPs identified by proteomic analysis. Using the "clusterProfiler" package in R, GO and KEGG enrichment analyses were conducted on the significant genes, with results visualized as bilateral symmetric bar charts using "ggplot2" and "patchwork" packages.

#### Clinical characteristics analysis

Data for the clinical characteristics analysis of LARS and PRIM2 were obtained from the GSE21257 dataset. 53 OS samples were first divided into a high-expression group (26 samples) and a low-expression group (27 samples) based on the median of gene expression levels. Then, clinical characteristics were organized into categorical variables: age was dichotomized into two groups (< = 15 years and > 15 years); gender was categorized into "Female" and "Male"; metastasis status was classified into "Metastasis" and "Non-metastasis"; histological subtypes were divided into "Chondroblastic", "Fibroblastic", "Osteoblastic", and others; Huvos grade (assessing tumor necrosis rate after neoadjuvant chemotherapy) was categorized into 1, 2, 3, 4, and "Unknown"; and survival status was classified into "Alive" and "Dead". Subsequently, the Chi-square test for clinical characteristics was performed using the "CreateTableOne" function from the R package "tableone" (version: 0.13.2) to determine whether there were significant differences in clinical characteristics between different sample groups, with a significance threshold of *p* < 0.05.

### Statistical analysis

GraphPad Prism 6.0 (USA, SCR_002798) was used to analyze group differences using a t-test for two groups and one-way ANOVA for three or more groups. Data were derived from at least three independent experiments and were presented as mean ± standard deviation (SD). Statistical significance is denoted as follows: *p < 0.05, **p < 0.01.

## Result

### LARS is highly expressed in primary OS and promotes proliferation while suppressing metastatic phenotypes

**Fig. 1:**
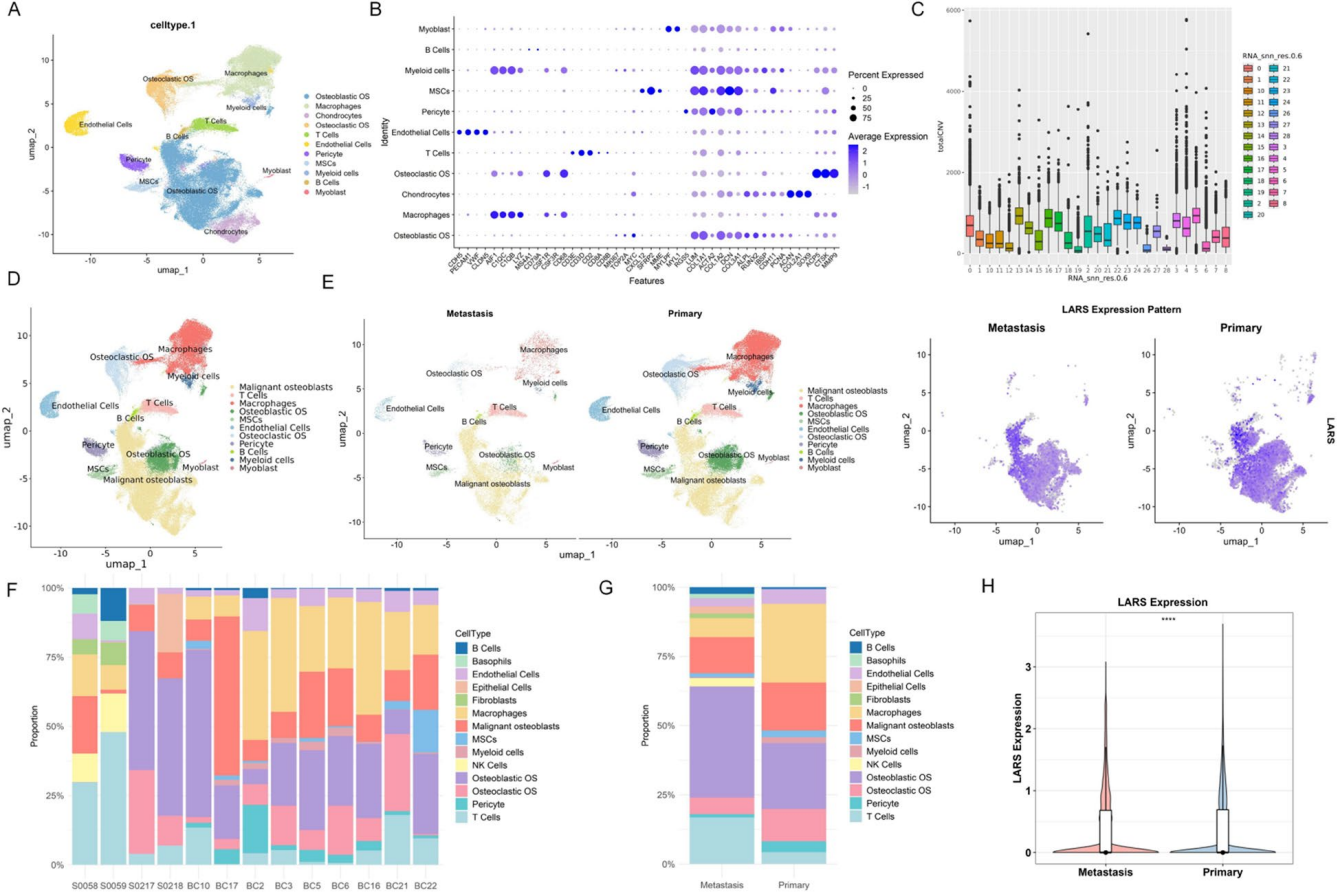
Single-cell transcriptomic landscape of OS and expression profiling of LARS in primary and metastatic lesions. **A** UMAP plot of scRNA-seq data (GSE152048) from 7 primary and 2 pulmonary metastatic OS samples. **B** Expression patterns of representative marker genes used for cell-type identification across different clusters. **C** Cell clustering was performed using the "FindClusters" function at a resolution of 0.6, yielding 29 clusters. Each cluster was subsequently evaluated for CNV scores, and those with high CNV levels were annotated as malignant cells. **D** UMAP visualization illustrating the overall single-cell transcriptomic architecture and interrelationships among identified cell clusters. **E** Comparative UMAP plots of primary and metastatic OS samples showing differences in cellular distribution. **F** Stacked bar plot showing the proportions of each cell type across 13 analyzed samples. Among these, BC2, BC3, BC5, BC6, BC16, BC21, and BC22 are primary lesions sourced from GSE152048; BC10 and BC17 are metastatic lesions from GSE152048; and S0058, S0059, S0217, S0218 are metastatic lesions sourced from GSE270231. **G** The mean cell-type proportions from 7 primary and 6 metastatic OS samples shown in Fig. 1F are summarized in Fig. 1G, allowing comparison of cellular composition between the two groups. **H** Targeting LARS expression in malignant osteoblasts and a UMAP feature plot illustrating the heterogeneous spatial distribution of LARS expression

Based on the scRNA-seq data from the GEO dataset GSE152048, comprising 7 primary and 2 pulmonary metastatic OS samples, we performed a comprehensive analysis to characterize gene expression differences between primary and metastatic lesions. After standard preprocessing steps including quality control, normalization, and principal component analysis, we constructed a cell neighbor graph and performed clustering, which identified 11 distinct cell populations. These were visualized in two dimensions using UMAP and annotated as osteoblastic OS, macrophages, chondrocytes, osteoclastic OS, T cells, endothelial cells, pericytes, MSCs, myeloid cells, B cells, and myoblasts (Fig. 1A). Cell type identification was further corroborated by examining the expression patterns of well-established marker genes (Fig. 1B). To distinguish malignant from normal cells, CNV analysis was performed on the osteoblastic subpopulation. Clusters with high CNV scores were annotated as malignant osteoblastic cells, while those with low scores were defined as normal. Chondrocytes exhibiting elevated CNV scores were also reclassified as malignant osteoblastic cells (Fig. 1C). The UMAP visualization effectively illustrated the global architecture of the single-cell transcriptome and the structure of the identified cell clusters (Fig. 1D). Comparative UMAP plots between primary and metastatic samples revealed differences in cellular distribution (Fig. 1E). To quantify the differences in cellular composition between primary and metastatic sites, we analyzed the proportional abundance of each cell type across the 9 samples using stacked bar plots. Samples S0058, S0059, S0217, S0218, BC10, and BC17 were derived from metastatic lesions, while the others were from primary sites (Fig. 1F). Aggregated comparison of cell-type proportions across primary versus metastatic samples revealed a higher fraction of osteoclastic OS cells in primary tumors than in metastases (Fig. 1G), suggesting a relatively osteolytic microenvironment in primary lesions that may facilitate tumor growth through bone matrix degradation. A detailed summary of cell-type proportions in primary and metastatic OS samples is provided in Table S2. In addition to the reduced abundance of osteoclastic OS cells, metastatic lesions exhibited a decrease in macrophages and malignant osteoblast-like cells. In contrast, the relative proportions of B cells and T cells were increased in metastatic samples compared with primary tumors, indicating a marked remodeling of the immune microenvironment during metastatic progression. Based on prior research, we identified LARS as crucial for the proliferation of OS via a comprehensive analysis of the large-scale CRISPR-Cas9 screening database [20]. To explore the potential role of the LARS in OS progression, we focused on its expression pattern within the malignant osteoblastic cells. Our analysis revealed that LARS expression was significantly higher in primary sites compared to metastatic sites (*p* < 0.05). Spatial visualization of LARS expression across the UMAP further suggested its heterogeneous distribution, underscoring its context-dependent expression within the tumor ecosystem (Fig. 1H).

**Fig. 2:**
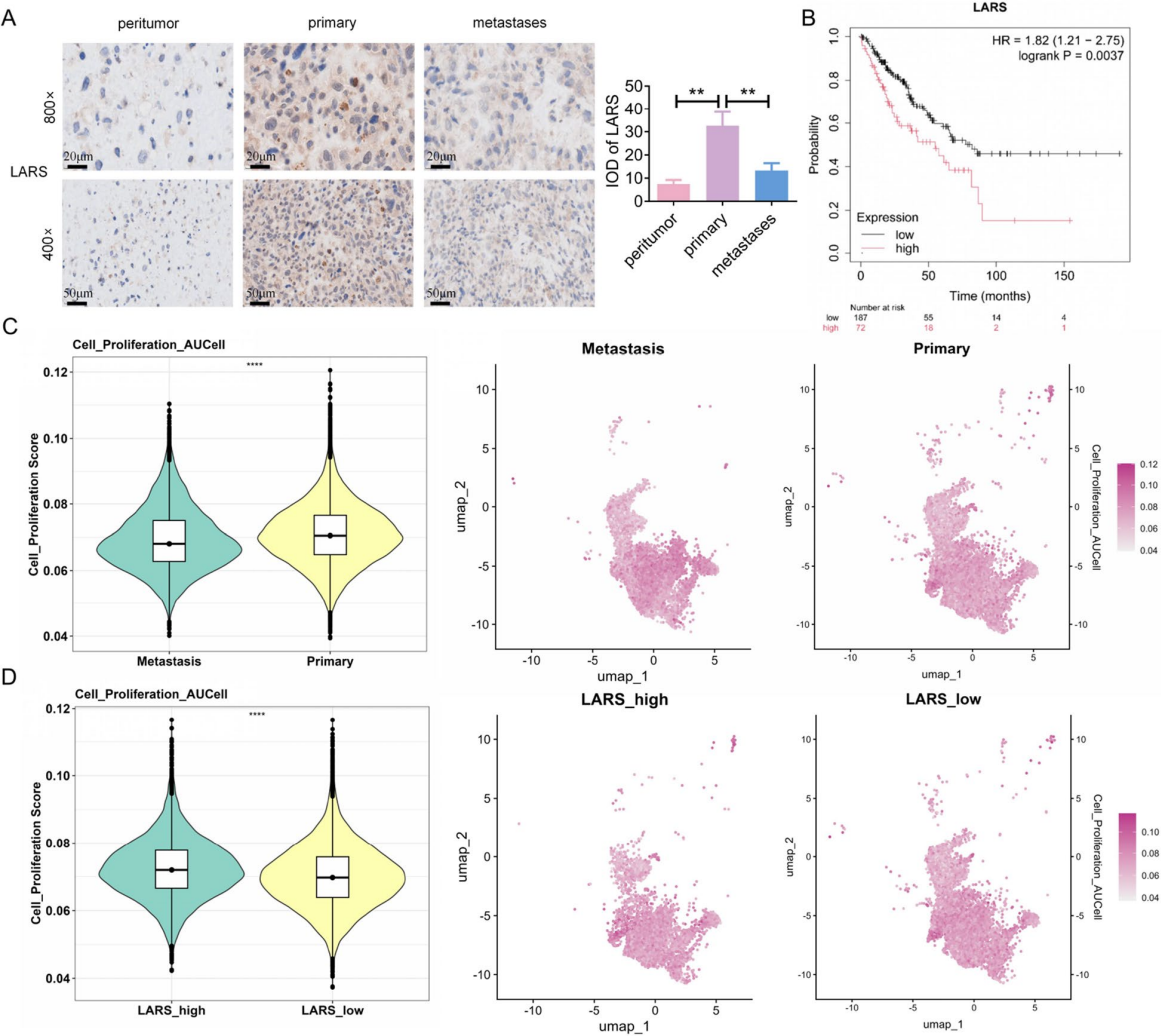
Analysis of LARS expression and its correlation with proliferative activity in OS. **A** IHC assays were performed to evaluate LARS expression in paired peritumoral, primary tumor, and synchronous metastatic tissues of OS samples (*n* = 8 paired peritumoral and primary samples; *n* = 6 paired synchronous metastatic lesions from the same patients; scale bar: 20 μm and 50 μm; **p < 0.01). **B** Survival analysis of overall survival in OS samples with high versus low LARS expression, using the Kaplan–Meier Plotter database. **C** Violin (left) and UMAP (right) plots showing proliferation activity scores of malignant osteoblastic cells from primary and metastatic OS samples. **D** Violin (left) and UMAP (right) plots illustrating differential distribution and proliferative activity between LARS-high and LARS-low malignant osteoblastic cells in primary OS samples

Moreover, clinical characteristic analysis data of LARS on OS showed that the proportion of metastatic patients was significantly higher in the low-expression group compared to the high-expression group (*p* = 0.029) (Table 5), indicating that low LARS expression may be associated with elevated metastatic risk in OS. Consistently, IHC staining confirmed that LARS expression was significantly higher in primary tumors than in both metastatic lesions and paracancerous tissue (Fig. 2A). Besides, survival analysis of LARS using the Kaplan–Meier Plotter displayed that patients with low LARS expression (*n* = 187) exhibited a significantly better overall survival than those with high expression (*n* = 72) (*p* = 0.0037) (Fig. 2B). We next assessed the proliferative potential of OS cells. A violin plot revealed that malignant osteoblastic cells from primary lesions displayed markedly higher proliferative activity than metastasis lesions, which was further visualized by UMAP mapping of proliferation scores (Fig. 2C). To explore the relationship between LARS expression and proliferation, malignant osteoblastic cells from primary tumors were divided into LARS-high and LARS-low groups. The LARS-high subgroup exhibited significantly greater proliferative capacity, as shown by both violin and UMAP plots (Fig. 2D), suggesting that LARS may contribute to enhanced proliferation of malignant osteoblasts in primary OS.

**Table 5:** Clinical characteristic analyses of LARS

Character	Level	Low expression of LARS	High expression of LARS	P	Test^b^
Age	< = 15 years	11 (42.3)^a^	10 (37.0)	0.911	χ^2^
	> 15 years	15 (57.7)	17 (63.0)		
Gender	Female	7 (26.9)	12 (44.4)	0.297	χ^2^
	Male	19 (73.1)	15 (55.6)		
Metastasis status	Metastasis	21 (80.8)	13 (48.1)	0.029	χ^2^
	Non-metastasis	5 (19.2)	14 (51.9)		
Histological subtype	Chondroblastic	4 (15.4)	2 (7.4)	0.741	Fisher
	Fibroblastic	2 (7.7)	3 (11.1)		
	Osteoblastic	16 (61.5)	16 (59.3)		
	others	4 (15.4)	6 (22.2)		
Huvos grade	1	9 (34.6)	4 (14.8)	0.402	Fisher
	2	7 (26.9)	9 (33.3)		
	3	6 (23.1)	7 (25.9)		
	4	1 (3.8)	4 (14.8)		
	Unknown	3 (11.5)	3 (11.1)		
Vital status	Alive	12 (46.2)	18 (66.7)	0.219	χ^2^
	Dead	14 (53.8)	9 (33.3)		

^a^N (X), where N represents the number of patients and X indicates the percentage of this item within the category

^b^Statistical tests: Categorical variables were compared using the χ^2^ test (Chi-square) or Fisher’s exact test when expected cell counts were < 5

**Fig. 3:**
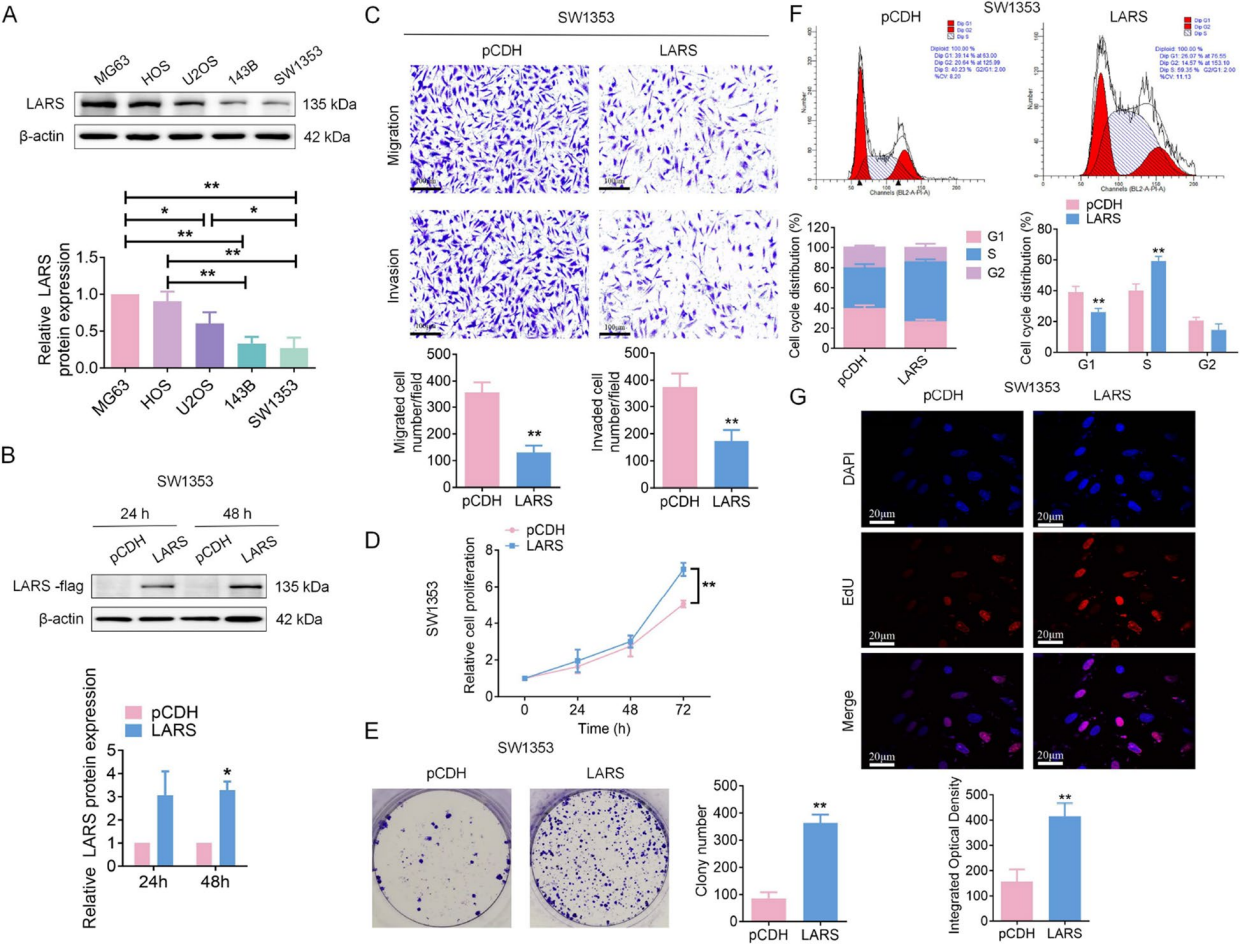
LARS is highly expressed in SW1353 cells and promotes malignant phenotypes. **A** WB assays were used to detect the expression level of LARS in OS cell lines (MG-63, HOS, U2OS, 143B, and SW1353; *n* = 3; *p < 0.05, **p < 0.01). **B** SW1353 cells were transfected with either a LARS-overexpressing recombinant plasmid (LARS) or an empty plasmid (pCDH), respectively. The transfection efficiency was then detected by WB (*n* = 3; *p < 0.05). **C** Transwell assays were used to examine the effect of LARS overexpression on the migration and invasion of SW1353 cells (scale bar: 100 μm; *n* = 3; *p < 0.05, **p < 0.01). **D** MTT assays were used to detect the effect of LARS overexpression on the proliferation of SW1353 cells (*n* = 3; **p < 0.01). **E** Colony formation assays were employed to investigate the effect of LARS overexpression on the colony formation of SW1353 cells (*n* = 3; **p < 0.01). **F** Flow cytometry was used to detect the effect of LARS overexpression on the cell cycle of SW1353 cells, which was evaluated immediately 48 h after transduction (*n* = 3; **p < 0.01). **G** EdU (red) and DAPI (blue) staining was used to assess the effect of LARS overexpression on the proliferation of SW1353 cells (magnification: 630 ×; scale bar: 20 μm; *n* = 3; **p < 0.01)

In terms of cell function, we first examined LARS baseline expression in OS cell lines MG63, HOS, U2OS, 143B, and SW1353, widely used in OS basic research due to their diverse genetic and pathological features [21, 22]. LARS expression was observed ubiquitously across all cell lines, with significantly higher levels detected in MG63 and HOS, and markedly lower levels in 143B and SW1353 (Fig. 3A). We next constructed LARS overexpression in SW1353 and 143B cells with low endogenous LARS expression, and verified the overexpression efficiency by WB analysis at 24 and 48 h post-transfection (Fig. 3B; Fig. S1A). 143B cells transfected for 24 h and SW1353 cells transfected for 48 h were selected for further experimental procedures. Notably, following LARS overexpression in OS cells significantly inhibited the migratory and invasive capabilities (Fig. 3C; Fig. S1B), while enhancing cell proliferation and colony-forming ability compared to the control cells (Fig. 3D-E; Fig. S1C-D). Cell cycle analysis revealed that LARS overexpression accelerated cell cycle progression by promoting the S phase entry in OS cells (Fig. 3F; Fig. S1E). To further confirm the proliferation results, EdU analysis revealed increased proliferation rates in the LARS-overexpressed OS cells (Fig. 3G; Fig. S1F).

Subsequently, we created LARS-silenced OS cell lines (MG-63 and HOS) using siRNA, and selected siR-LARS-2 for further experiments due to its highest silencing efficiency (Fig. S2A; Fig. S3A), with MG-63 and HOS cells harvested 48 h post-transfection for subsequent procedures. The result demonstrated that LARS silencing in OS cells led to enhanced migration and invasion, alongside diminished proliferation and clonogenic capacity (Fig. S2B-D; Fig. S3B-D). Additionally, LARS silencing in OS cells resulted in cell cycle arrest (Fig. S2E; Fig. S3E). EdU assays further confirmed decreased cell proliferation in the LARS-silenced OS cells (Fig. S2F; Fig. S3F). These results suggest the crucial role of LARS in OS cell behavior.

### LARS overexpression enhances glycolytic metabolism, promotes DNA replication, and mitigates ER stress in OS cells

**Fig. 4:**
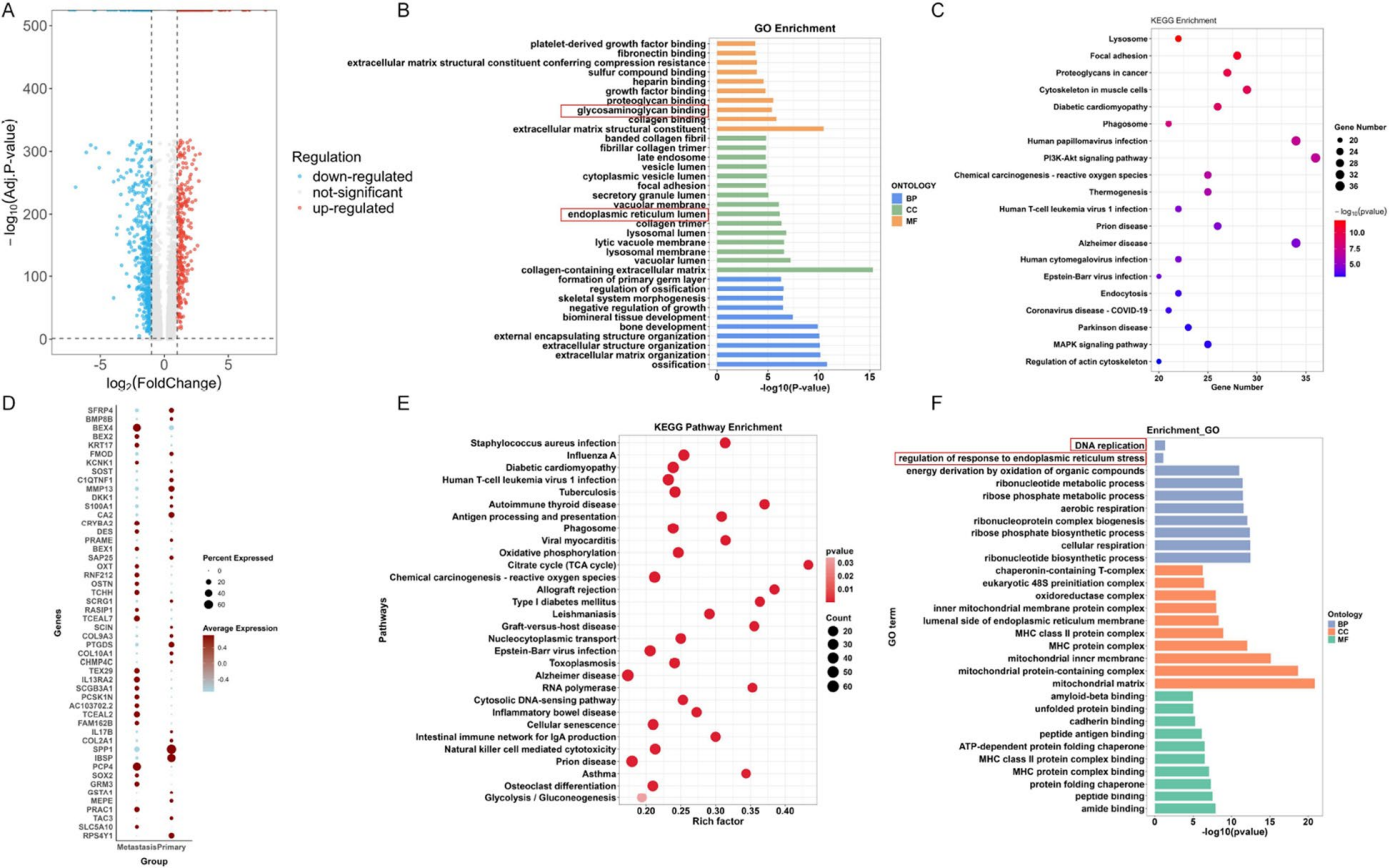
Bioinformatics analysis identifies glycolysis as a key process associated with OS metastasis. **A** Volcano plot displaying the overall distribution of DEGs between primary and metastatic OS lesions, with 526 genes upregulated and 633 genes downregulated in metastatic tumors. **B** GO enrichment analysis of DEGs showing significant enrichment in biological processes. **C** KEGG pathway enrichment analysis of DEGs, highlighting the significantly enriched signaling pathways associated with OS metastasis. **D** Bubble plot presenting the top 50 most significantly altered genes ranked by *p*-value. (E&F) The biological processes and signaling pathways in which DEGs were enriched were identified through the KEGG (**E**) and GO (**F**) enrichment analysis

**Fig. 5:**
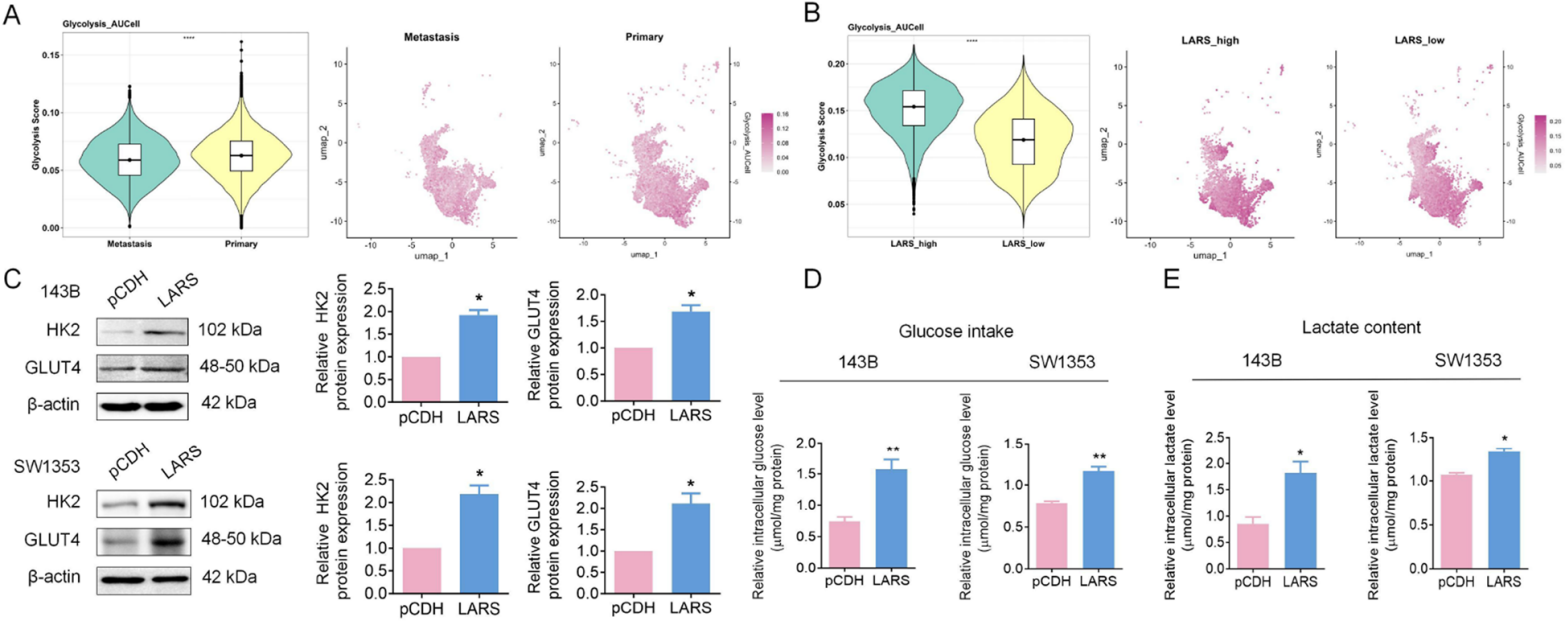
LARS overexpression promotes glycolysis in OS cells. **A** Violin (left) and UMAP (right) plots showing glycolytic activity of malignant osteoblastic cells from primary and metastatic OS samples. **B** Violin (left) and UMAP (right) plots illustrating differences in glycolytic activity between LARS-high and LARS-low malignant osteoblastic cells in primary OS samples. **C** WB assays were used to detect the effect of LARS overexpression on the expression levels of glycolysis-related proteins, including HK2 and GLUT4, in 143B and SW1353 cells (*n* = 3; *p < 0.05). (D&E) The effect of LARS overexpression on glucose intake (D) and lactate content (E) in 143B and SW1353 cells was measured using glucose and lactate assay kits (*n* = 3; **p < 0.01)

To further elucidate the molecular differences between primary and metastatic OS lesions, differential expression analysis was performed between the two groups, identifying 526 upregulated and 633 downregulated genes. The overall distribution of DEGs was visualized using a volcano plot (Fig. 4A). GO and KEGG enrichment analyses of these DEGs revealed significant enrichment in biological processes and pathways related to glycosaminoglycan binding and the endoplasmic reticulum lumen (Fig. 4B-C). Moreover, a bubble plot of the top 50 most significant DEGs (ranked by *p*-value) highlighted that SPP1 (Secreted Phosphoprotein 1) and IBSP (Integrin-Binding Sialoprotein) were predominantly upregulated in primary lesions, whereas BEX4 (Brain Expressed X-Linked 4) and PCP4 (Purkinje Cell Protein 4) were more highly expressed in metastatic lesions (Fig. 4D), suggesting their potential involvement in OS progression and metastatic dissemination. Building upon these findings, to explore the potential mechanisms underlying LARS-mediated promotion of proliferation and inhibition of metastasis in OS, we analyzed DEGs between LARS-high and LARS-low expression groups using TCGA dataset-derived OS data. GO and KEGG enrichment analysis revealed that LARS-related DEGs participate in multiple cancer-related pathways in OS, including glycolysis metabolism, the intricate process of DNA replication, and the regulation of ER stress responses (Fig. 4E-F). We first investigated the association between LARS expression and glycolytic activity in OS cells using scRNA-seq data. The analysis revealed markedly elevated glycolysis scores in primary tumors, with LARS-high OS cells displaying even greater glycolytic activity, as further illustrated by UMAP visualization (Fig. 5A-B). Subsequently, the expression of glycolytic markers hexokinase 2 (HK2) and glucose transporter 4 (GLUT4) was examined in OS cells following LARS overexpression. The results showed increased protein levels of both markers (Fig. 5C), accompanied by increased intracellular glucose intake and lactate production (Fig. 5D-E). These results suggest that LARS plays a certain role in the metabolic reprogramming of OS cells, potentially promoting cell proliferation rather than motility [23].

**Fig. 6:**
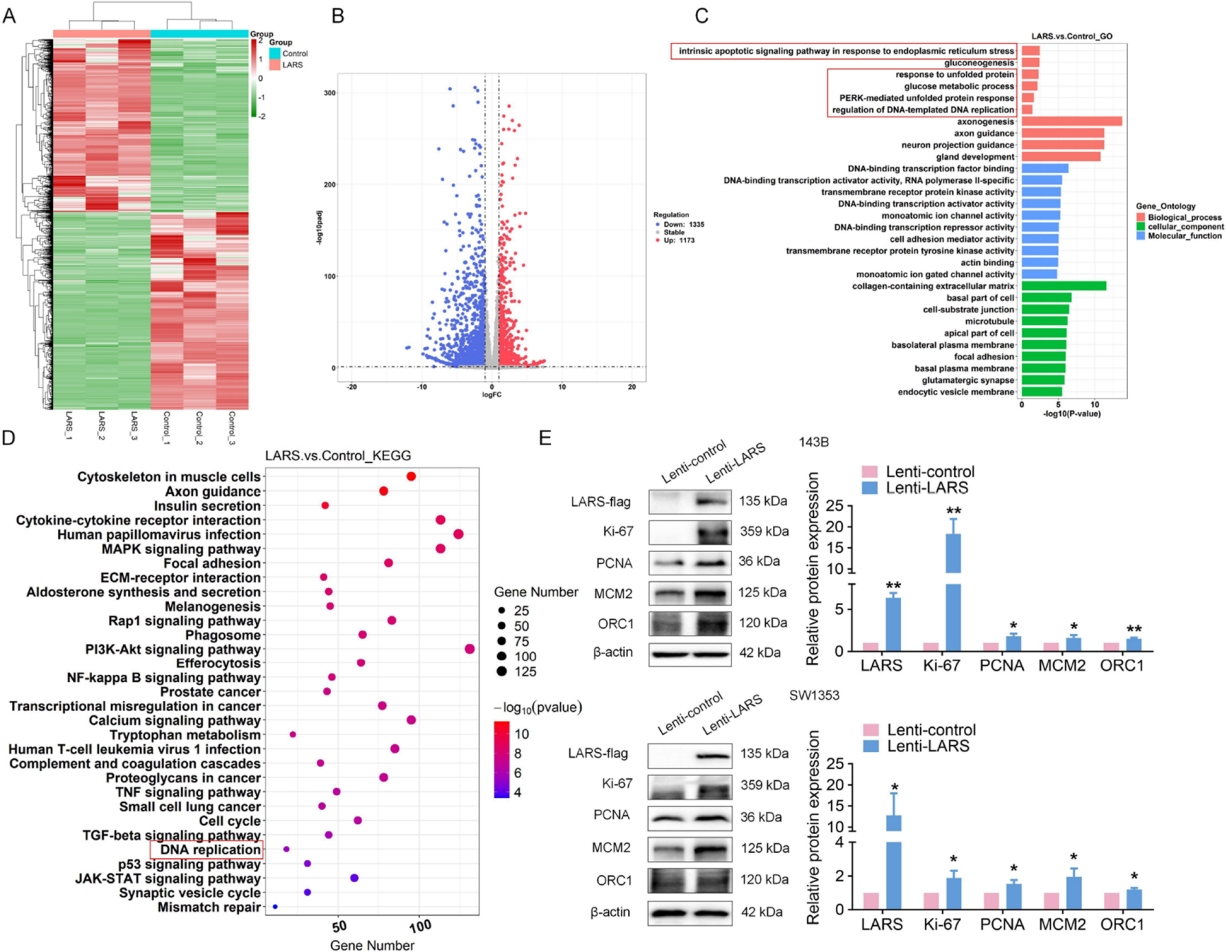
LARS overexpression promotes DNA replication and cell cycle progression in OS. **A** The heatmap was used to visualize the DEGs from transcriptome sequencing between SW1353 cells stably transfected with LARS overexpression and control cells. **B** The DEGs between the two groups were analyzed, and their distribution was depicted using a volcano plot. **C** and **D** The biological processes and signaling pathways in which DEGs were enriched were identified by GO (**C**) and KEGG (**D**) enrichment analysis. **E** WB assays were used to detect the expression levels of Ki-67, PCNA, MCM2, and ORC1 in 143B and SW1353 cells transfected with Lenti-LARS or Lenti-control (*n* = 3; *p < 0.05, **p < 0.01)

To gain a more comprehensive insight into the role of LARS in OS cells, we performed transcriptome sequencing on SW1353 cells that were stably transfected with LARS overexpression and their corresponding control cells. The heat map illustrated the DEGs (Fig. 6A), and the volcano plot distinctly exhibited the separate upregulation (1173 genes) and downregulation (1335 genes) of these DEGs (Fig. 6B). GO and KEGG analyses of these DEGs revealed enrichment in pathways such as glucose metabolic process, DNA replication, cell cycle, unfolded protein and ER stress responses (Fig. 6C-D). Furthermore, the overexpression of LARS in OS cells upregulated the markers associated with proliferation and DNA replication, including Ki-67 and proliferating cell nuclear antigen (PCNA) (Fig. 6E), both of which are critical in cell division and DNA synthesis or repair [24–26], and were significantly increased. Additionally, the expression of minichromosome maintenance complex component 2 (MCM2) and origin recognition complex subunit 1 (ORC1), both of which are integral components of the pre-replication complex essential for initiating DNA replication [27], was also elevated. These findings support sequencing results highlighting the importance of DNA replication in cancer progression [28, 29].

**Fig. 7:**
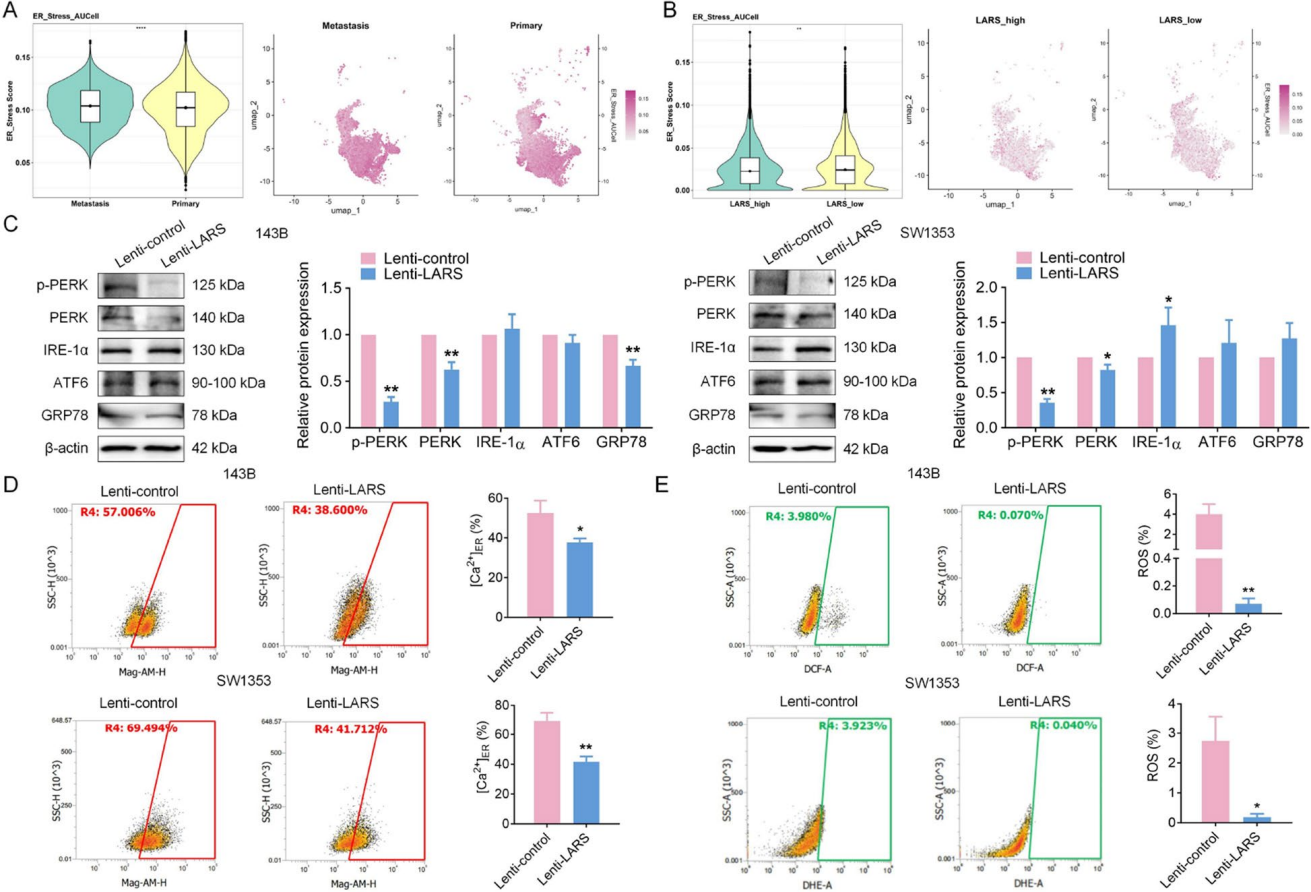
LARS overexpression alleviates ER stress in OS. **A** Violin (left) and UMAP (right) plots showing the ER stress scores of malignant osteoblastic cells derived from primary and metastatic OS samples. **B** Violin (left) and UMAP (right) plots illustrating differential ER stress scores between LARS-high and LARS-low malignant osteoblastic cells in primary OS samples. **C** WB assays were used to detect the expression levels of p-PERK, PERK, IRE-1α, ATF6, and GRP78 in 143B and SW1353 cells transfected with Lenti-LARS or Lenti-control (*n* = 3; *p < 0.05, **p < 0.01). **D** Flow cytometry assays were used to detect the ER calcium ion concentration level in 143B and SW1353 cells transfected with Lenti-LARS or Lenti-control (*n* = 3; *p < 0.05, **p < 0.01). **E** Flow cytometry assays were used to detect the level of ROS in 143B and SW1353 cells transfected with Lenti-LARS or Lenti-control (*n* = 3; *p < 0.05, **p < 0.01)

Concurrently, based on scRNA-seq data, we evaluated the ER stress levels of malignant osteoblastic cells. The results showed that ER stress scores were relatively lower in primary lesions compared to metastatic sites, and within primary malignant osteoblasts, the LARS-high subgroup exhibited reduced ER stress scores compared to the LARS-low subgroup, as visualized by UMAP (Fig. 7A-B). We further evaluated the expression of proteins associated with ER stress-related factors [30], including endoplasmic reticulum stress kinase PERK (EIF2AK3), phosphorylated PERK (p-PERK), inositol-requiring enzyme 1 alpha (IRE-1α), activating transcription factor 6 (ATF6), and glucose-regulated protein 78 (GRP78), in OS cells with stable overexpression of LARS. Notably, PERK expression was significantly downregulated in LARS-overexpressing OS cells. While the reduction was more pronounced in 143B cells, a modest but statistically significant decrease was also observed in SW1353 cells, suggesting a cell line-dependent degree of modulation. This downregulation was further supported by a consistent reduction in p-PERK across both cell lines, suggesting a potential modulation of the unfolded protein response (UPR) pathways, which may contribute to the alleviation of ER stress [31]. Intriguingly, the expression levels of IRE-1α, ATF6, and GRP78 did not exhibit statistically significant or stable alterations in both cell lines simultaneously (Fig. 7C), implicating the potential involvement of other pathways in ER stress. Given the crucial role of Ca^2+^ and ROS in DNA replication and ER stress [32–35], we observed decreased Ca^2+^ and ROS levels in LARS-overexpressing OS cells compared to controls (Fig. 7D-E). To further validate our findings, we performed rescue experiments on LARS-overexpressing OS cells, examining their responses following treatment with CCT020312, a PERK activator [36]. By employing a range of CCT020312 concentrations, we observed a dose-dependent inhibitory effect of this compound on the survival of OS cells (Fig. S4A; Fig. S5A). As expected, the integration of LARS-overexpression in OS cells with CCT020312 treatment significantly reduced the stimulatory effect of LARS on cell proliferation (Fig. S4B; Fig. S5B). This combination also effectively reversed the inhibitory impact of LARS on the levels of ROS (Fig. S4C; Fig. S5C), the concentration of Ca^2+^ within the ER (Fig. S4D; Fig. S5D), and the expression of PERK-related downstream factors associated with ER stress [37], including phosphorylated PERK (p-PERK), activating transcription factor 4 (ATF4), and C/EBP homologous protein (CHOP) (Fig. S4E; Fig. S5E), demonstrating that LARS overexpression regulates calcium homeostasis and ROS levels to mediate DNA replication and PERK-related ER stress in OS cells. Another rescue experiment further validated our conclusions. Flow cytometry results revealed that LARS knockdown promoted ROS accumulation, while ROS levels were reduced upon treatment with the ROS scavenger NAC (Fig. S4F; Fig. S5F). Consistently, WB analysis confirmed that LARS knockdown upregulated the expression of p-PERK, ATF4, and CHOP. Notably, the activation of the ER stress signaling pathway induced by LARS knockdown was markedly alleviated upon NAC treatment (Fig. S4G; Fig. S5G). Collectively, these rescue experiments provide evidence that LARS modulates OS cell proliferation by maintaining redox and calcium homeostasis, thereby suppressing PERK-mediated ER stress activation.

### LARS overexpression enhances primary tumor growth while inhibiting metastasis* in vivo*

**Fig. 8:**
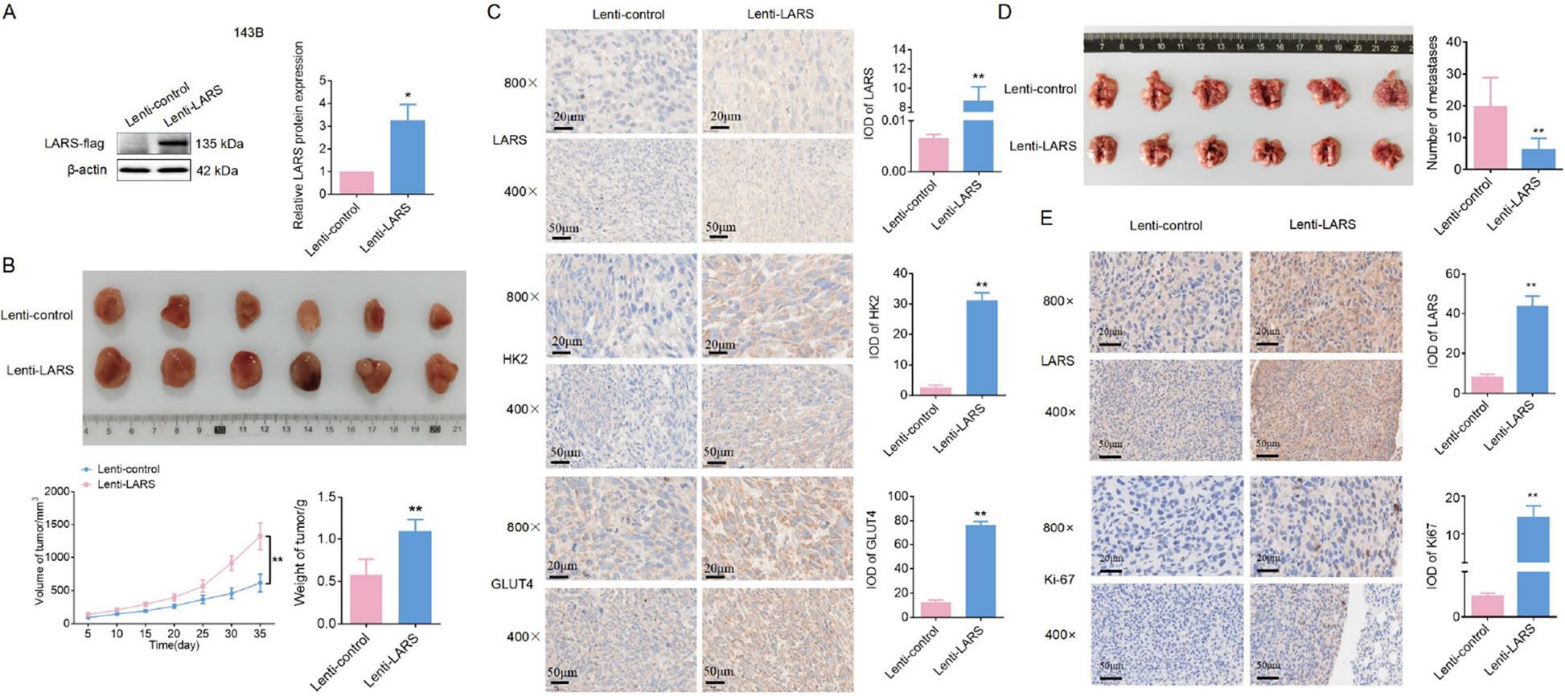
LARS overexpression promotes tumor proliferation while inhibiting metastasis *in vivo*. **A** 143B cells were transfected with either Lenti-LARS or Lenti-control vectors. The transfection efficiency was assessed using WB assays (*n* = 3; *p < 0.05). **B** The nude mice were subcutaneously injected with LARS-stably overexpressing or control 143B cells (5 × 10⁶/200 μL). Representative images of xenograft tumors, along with measurements of tumor volume and weight, were recorded (*n* = 6; **p < 0.01). **C** The expression levels of LARS, HK2, and GLUT4 in Lenti-control or Lenti-LARS tumor tissues were detected by the IHC assays (magnification: 200 × or 400 ×; *n* = 3; scale bar: 20 μm or 50 μm; **p < 0.01). **D** Lung metastasis assay. Mice were intravenously injected via the tail vein with K7M2 cells transfected with Lenti-LARS or Lenti-control vectors to establish lung metastases. The number of metastatic nodules on the lung surface was quantified (*n* = 6; **p < 0.01). (E) IHC analysis detects the expression of LARS, PRIM2, and Ki-67 in lung metastatic foci (magnification: 200 × or 400 ×; *n* = 3; scale bar: 20 μm or 50 μm; **p < 0.01)

To further investigate the *in vivo* effects of LARS, we established an OS xenograft tumor model using stable LARS-overexpressing 143B cells (Lenti-LARS), and verified the overexpression efficiency of LARS in the transfected cells by WB (Fig. 8A). Monitoring the weight and volume of xenograft tumors indicated that LARS-overexpressing cells exhibited accelerated growth compared to the control group (Fig. 8B), suggesting a pro-tumorigenic role for LARS *in vivo*. The IHC results further supported our findings, demonstrating elevated levels of LARS, HK2, and GLUT4 protein in LARS-overexpressing tumors (Fig. 8C). Furthermore, the *in vivo* impact of LARS knockdown was validated using MG-63 (Lenti-sh-LARS). WB analysis confirmed successful LARS knockdown in the xenograft model (Fig. S6A). Conversely to the tumor-promoting effects observed in LARS-overexpressing cohorts, LARS knockdown resulted in significantly reduced tumor volume and weight compared to the control (Fig. S6B). Moreover, a lung metastasis model was established by intraperitoneally injecting K7M2 cells with stable LARS overexpression, whose efficiency was validated by WB (Fig. S6C), and the results showed that LARS overexpression markedly suppressed metastatic capacity, as evidenced by the reduced number of pulmonary nodules compared with the control group (Fig. 8D). Consistent with its pro-proliferative role, IHC analysis revealed concomitant upregulation of LARS and Ki-67 in LARS-overexpressing metastases (Fig. 8E). Together, these *in vivo* experiments demonstrate that LARS exerts a dual role in OS progression, enhancing primary tumor growth while simultaneously restraining metastatic potential.

### LARS regulates proliferation and DNA replication in OS cells via PRIM2 upregulation

**Fig. 9:**
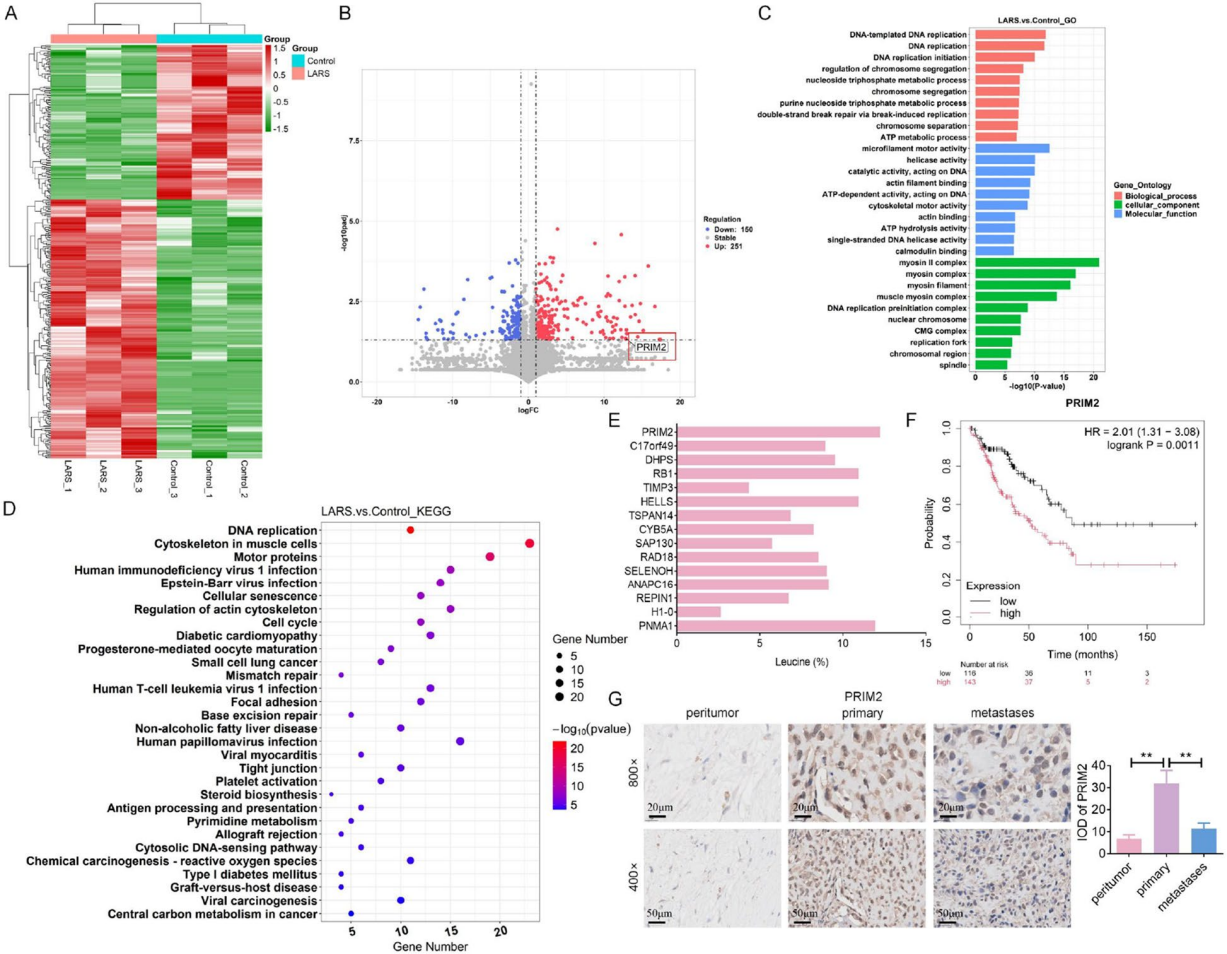
LARS enhances the expression levels of PRIM2 in OS as revealed by integrated proteomic and clinical analyses. **A** The heatmap was used to visualize the DEPs from proteomic profiling between SW1353 cells stably transfected with LARS overexpression and its corresponding control cells. **B** The DEPs between two groups were analyzed and their distribution was depicted using a volcano plot. (C&D) The biological processes and signaling pathways in which DEGs were enriched were identified by GO (**C**) and KEGG (**D**) enrichment analysis. **E** The Expasy website (https://www.expasy.org/) calculated the leucine residue proportion in the top 15 proteins with differential expression, selected from proteomic data screening. **F** Survival analysis of overall survival in OS samples with high versus low PRIM2 expression, using the Kaplan–Meier Plotter database. (G) The expression level of PRIM2 in peritumor (*n* = 8), primary (*n* = 8), and metastatic tissues (*n* = 6) was examined by the IHC assays (magnification: 800 × or 400 ×; scale bar: 20 μm & 50 μm; **p < 0.01)

A comprehensive proteomic analysis was conducted on SW1353 cells with stable overexpression of LARS compared to controls. This analysis yielded a heatmap illustrating differential protein expression (Fig. 9A) and a volcano plot showing the statistical significance of proteins related to LARS, identifying 251 upregulated and 150 downregulated proteins (Fig. 9B). GO and KEGG analysis annotated the functions of DEPs in LARS-overexpressing SW1353 cells, highlighting pathways to DNA replication and its initiation (Fig. 9C-D). Considering the role of LARS as a leucine tRNA synthetase, a histogram was generated to display the protein profile data for the top 15 DEPs, highlighting the proportion of leucine residues. Notably, PRIM2 was identified due to its high leucine residue ratio (Fig. 9E), which is critical for DNA replication processes [38]. Moreover, clinical characteristic analysis of PRIM2 in OS revealed a significant difference in Huvos grade between groups (*p* = 0.013), with a higher proportion of grade 2 patients in the high-expression group, suggesting that PRIM2 expression may be associated with the histological response to chemotherapy (Table 6). Characteristic analysis of PRIM2 expression suggested that higher PRIM2 expression was associated with poorer overall survival compared to low expression (*p* = 0.0011) (Fig. 9F). Besides, PRIM2 expression in clinical samples was assessed by IHC, showing a tendency toward higher PRIM2 levels in primary tumor tissues compared with metastatic tumor tissues (Fig. 9G).

**Table 6:** Clinical characteristic analyses of PRIM2

Character	Level	Low expression of PRIM2	High expression of PRIM2	P	Test^b^
Age	< = 15 years	9 (34.6)^a^	12 (44.4)	0.652	χ^2^
	> 15 years	17 (65.4)	15 (55.6)		
Gender	Female	8 (30.8)	11 (40.7)	0.638	χ^2^
	Male	18 (69.2)	16 (59.3)		
Metastasis status	Metastasis	17 (65.4)	17 (63.0)	1	χ^2^
	Non-metastasis	9 (34.6)	10 (37.0)		
Histological subtype	Chondroblastic	1 (3.8)	5 (18.5)	0.134	Fisher
	Fibroblastic	1 (3.8)	4 (14.8)		
	Osteoblastic	19 (73.1)	13 (48.1)		
	others	5 (19.2)	5 (18.5)		
Huvos grade	1	11 (42.3)	2 (7.4)	0.013	Fisher
	2	3 (11.5)	13 (48.1)		
	3	7 (26.9)	6 (22.2)		
	4	2 (7.7)	3 (11.1)		
	Unknown	3 (11.5)	3 (11.1)		
Vital status	Alive	13 (50.0)	17 (63.0)	0.5	χ^2^
	Dead	13 (50.0)	10 (37.0)		

^a^N (X), where N represents the number of patients and X indicates the percentage of this item within the category

^b^Statistical tests: Categorical variables were compared using the χ^2^ test or Fisher’s exact test when expected cell counts were < 5

**Fig. 10:**
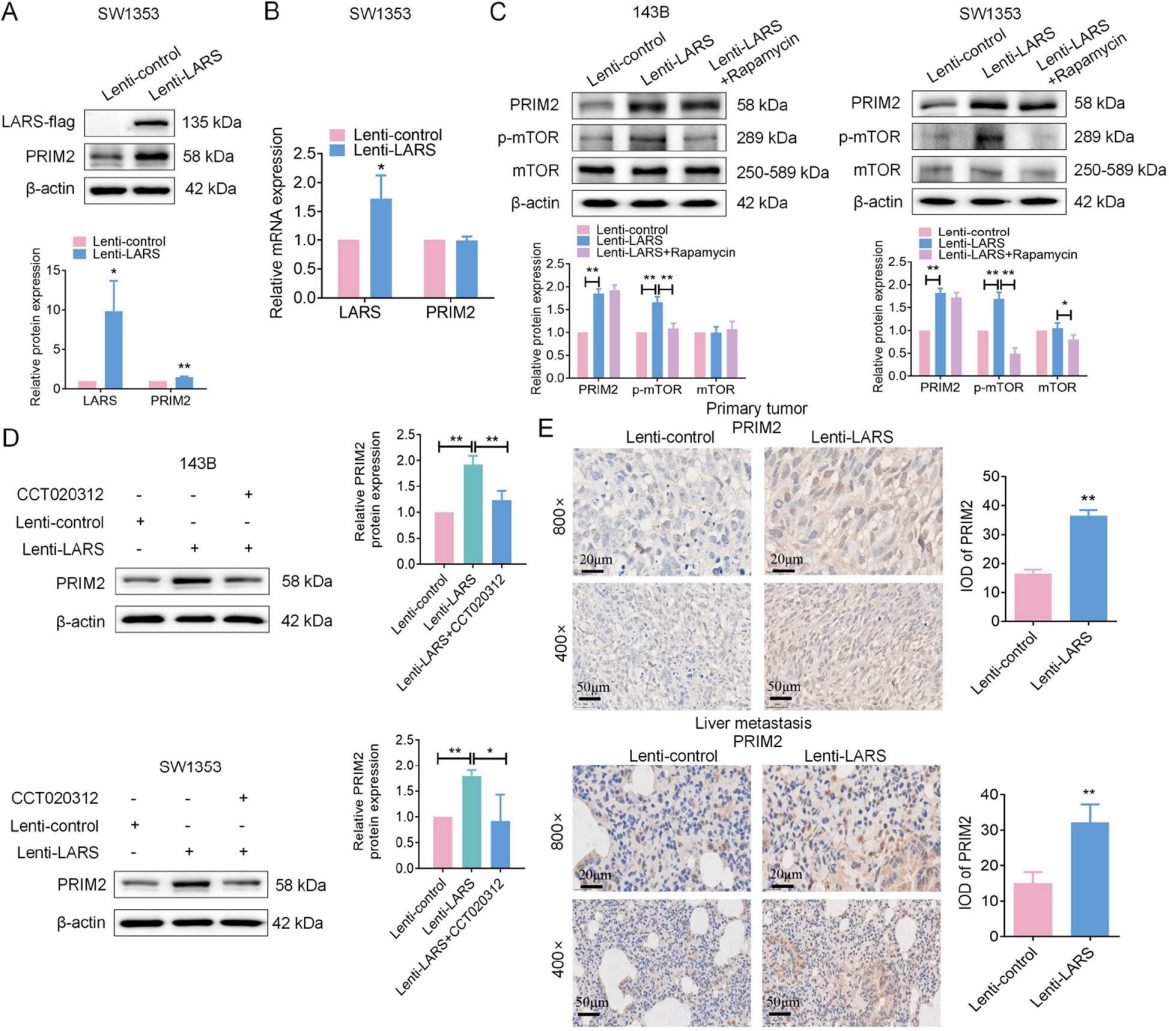
LARS upregulates PRIM2 expression via the mTOR signaling pathway. **A** WB assays were used to detect the expression level of PRIM2 in SW1353 cells transfected with Lenti-LARS or Lenti-control (*n* = 3; *p < 0.05, **p < 0.01). (B) RT-qPCR assays were used to detect the mRNA expression level of PRIM2 in SW1353 cells with LARS transfected with Lenti-LARS or Lenti-control. **C** WB assays were used to assess the effect of LARS stable overexpression combined with Rapamycin treatment on the expression level of PRIM2, p-mTOR, and mTOR in 143B and SW1353 cells (*n* = 3; *p < 0.05, **p < 0.01). (D) WB assays were used to assess the effect of LARS stable overexpression combined with CCT020312 treatment on the expression level of PRIM2 in 143B and SW1353 cells (*n* = 3; *p < 0.05, **p < 0.01). (E) The expression level of PRIM2 in Lenti-control or Lenti-LARS tumor tissues (from Fig. 8B & D) was examined using the IHC assays (magnification: 800 × or 400 ×; *n* = 6; scale bar: 20 μm or 50 μm; **p < 0.01)

Based on clinical observations, we further explored the potential relationship between PRIM2 and LARS in OS cell models. WB analysis showed increased PRIM2 protein levels in LARS-overexpressing SW1353 cells (Fig. 10A), whereas PCR analysis revealed no significant change in PRIM2 mRNA levels (Fig. 10B), suggesting that LARS may regulate PRIM2 at a post-transcriptional level. Considering the reported involvement of LARS in mTOR pathway activation [39], we examined whether LARS-associated PRIM2 upregulation was dependent on this pathway. PRIM2 protein levels in the Lenti-LARS combined with Rapamycin group were comparable to those in the Lenti-LARS group, suggesting that LARS-mediated PRIM2 regulation may occur independently of mTOR pathway activation (Fig. 10C). Furthermore, CCT020312 treatment reversed LARS-mediated PRIM2 upregulation in OS cells (Fig. 10D), implying a possible link between LARS-induced ER stress and PRIM2 regulation. Consistently, IHC analysis showed increased PRIM2 expression in both primary and lung metastatic tumors derived from LARS-overexpressing OS cells (Fig. 10E), supporting an association between LARS expression and PRIM2 upregulation *in vivo*.

**Fig. 11:**
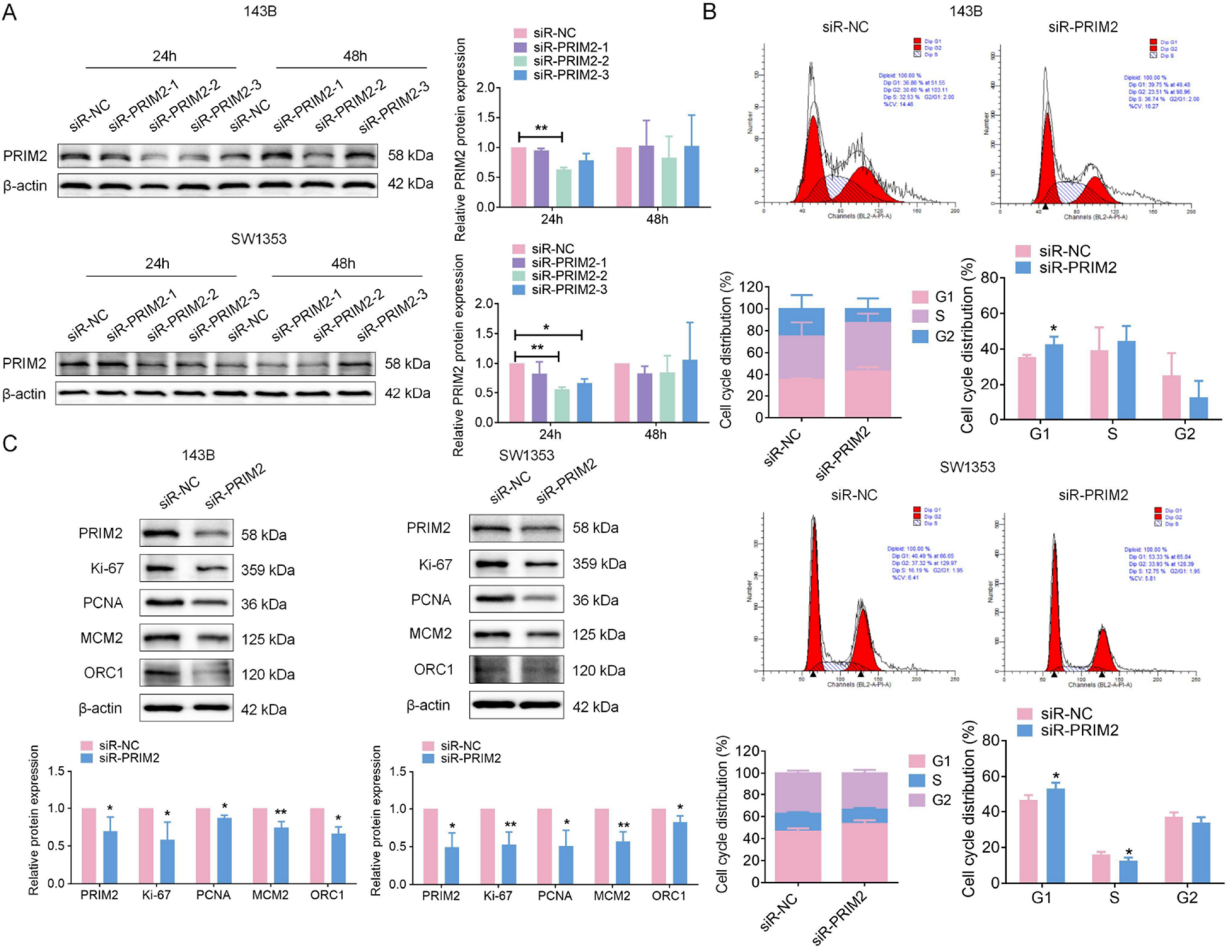
Knockdown of PRIM2 suppresses cell proliferation and DNA replication in OS cells. **A** WB analysis was used to detect knockdown efficiency at three LARS loci in 143B and SW1353 cells. (*n* = 3; *p < 0.05, **p < 0.01). **B** Flow cytometry assays were used to detect the effect of PRIM2 knockdown on the cell cycle of 143B and SW1353 cells, which was evaluated immediately 24 h after transfection (*n* = 3; *p < 0.05). **C** WB assays were used to assess the effect of PRIM2 knockdown on the expression levels of Ki-67, PCNA, MCM2, and ORC1 in 143B and SW1353 cells, with β-actin as the loading control (*n* = 3; *p < 0.05, **p < 0.01)

Given the crucial role of PRIM2 in promoting DNA replication and cancer progression, as documented in previous studies [19], we hypothesized that LARS may affect DNA replication and tumor cell proliferation through PRIM2 modulation. To verify this hypothesis, three siRNA targeting PRIM2 were designed, and PRIM2-2 showed the most efficient knockdown at 24 h post-transfection (Fig. 11A). Subsequent analyses indicated that PRIM2 silencing was associated with cell cycle arrest (Fig. 11B) and reduced expression of proliferation- and replication-related markers, including Ki-67, PCNA, MCM2 and ORC1 (Fig. 11C). Functional assays further showed that PRIM2 knockdown partially reversed the proliferative and cell cycle-promoting effects of LARS overexpression in SW1353 (Fig. S7 A-C) and 143B cells (Fig. S8 A-C). These effects were also observed at the protein level for Ki-67, PCNA, MCM2, and ORC1 (Fig. S7D; Fig. S8D). We then established SW1353 cells with PRIM2 overexpression to investigate the impact of PRIM2 upregulation on LARS in OS cells. Cells transfected for 24 h were selected for further experimentation due to their optimal overexpression efficiency (Fig. S9A). As anticipated, PRIM2 overexpression combined with LARS knockdown partially reversed the proliferation and cell cycle inhibition observed with siR-LARS alone in SW1353 cells (Fig. S9 B-D). Similarly, PRIM2 overexpression could modulate the expression of those proliferation and replication markers to counteract the inhibitory effects of siR-LARS at the protein level for Ki-67, PCNA, MCM2, and ORC1 detection (Fig. S9E). Together, these findings suggest that PRIM2 may function as a downstream mediator of LARS in regulating OS cell proliferation and DNA replication, although additional studies are required to further elucidate the underlying mechanisms.

### LARS-mediated leucine consumption enhances PRIM2 translation and modulates metabolic and malignant phenotypes in OS cells

**Fig. 12:**
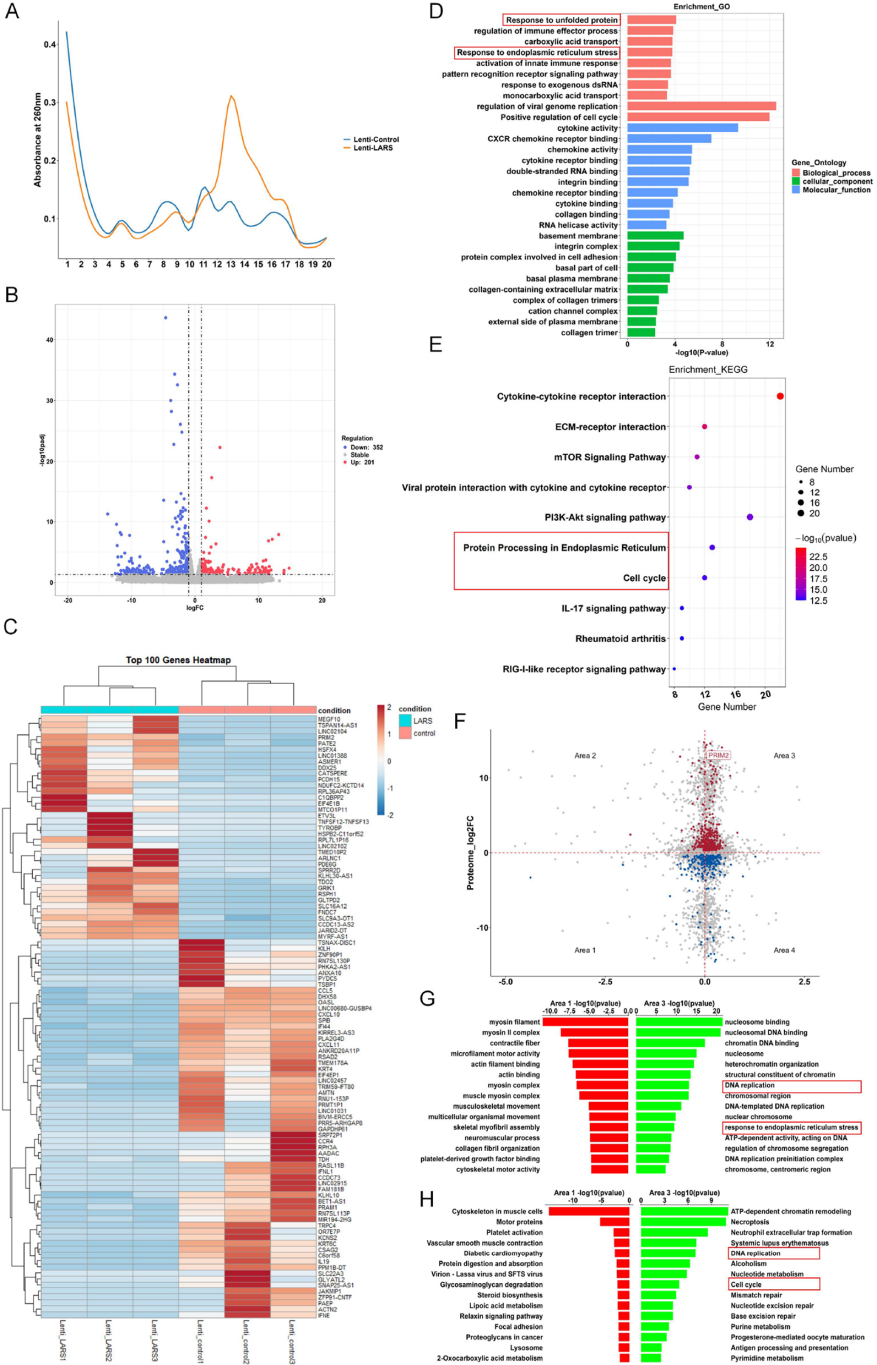
Multi-omics analysis reveals that LARS regulates mRNA translation. **A** Sucrose density gradient centrifugation analysis and the absorbance values of mRNA in 20 tubes were performed on SW1353 cells with stable overexpression of LARS or control. **B** The DEGs from the mRNA-Seq of sucrose density gradient centrifugation products of SW1353 cells stable overexpression of LARS or control were depicted using a volcano plot. **C** The heatmap was used to visualize the DEGs from the sequencing of sucrose density gradient centrifugation products of SW1353 cells stably overexpressing LARS or control. (D&E) The biological processes and signaling pathways in which DEGs were enriched were identified by GO (**D**) and KEGG (**E**) enrichment analysis. **F** A dot plot was created to show the overlap between DEGs from mRNA-Seq and DEPs identified by proteomic analysis. **G** and **H** Proteomic analysis of DEPs and mRNA-Seq of DEGs from sucrose density gradient centrifugation products were performed on SW1353 cells with stable overexpression of LARS or control, followed by GO (**G**) and KEGG (**H**) enrichment of regions 1 and 3

As LARS is vital for leucine transport and ribosomal protein synthesis [40], we further investigated the underlying mechanisms of LARS in the regulation of cell metabolism and gene expression in OS cells. The analysis of polyribosomes using sucrose density gradient centrifugation revealed that SW1353 cells, which stably overexpressed LARS, exhibited a peak in A260 absorption specifically in the fraction collected from tube number 13 (Fig. 12A). This finding prompted us to perform mRNA-Seq on these samples for further analysis. The sequencing results identified 201 upregulated and 352 downregulated DEGs compared with the control group, which were visualized using a volcano plot (Fig. 12B) and a heatmap displaying the top 100 genes (Fig. 12C). KEGG and GO enrichment analyses indicated that these DEGs were mainly enriched in the cell cycle, protein processing in the endoplasmic reticulum, and unfolded protein responses (Fig. 12D-E). By overlapping DEGs from mRNA-Seq with DEPs identified through proteomic profiling, we identified 6,490 intersecting elements (gray dots). Among these, 262 were significantly upregulated in both (red dots, including PRIM2), and 94 were significantly downregulated in both (blue dots) (Fig. 12F). Subsequent GO enrichment analysis showed that genes significantly upregulated in both mRNA-Seq and proteomic profiling were notably enriched in DNA replication and the response to ER stress (Fig. 12G). KEGG analysis further indicated the enrichment of these genes in biological pathways such as DNA replication and the cell cycle (Fig. 12H).

**Fig. 13:**
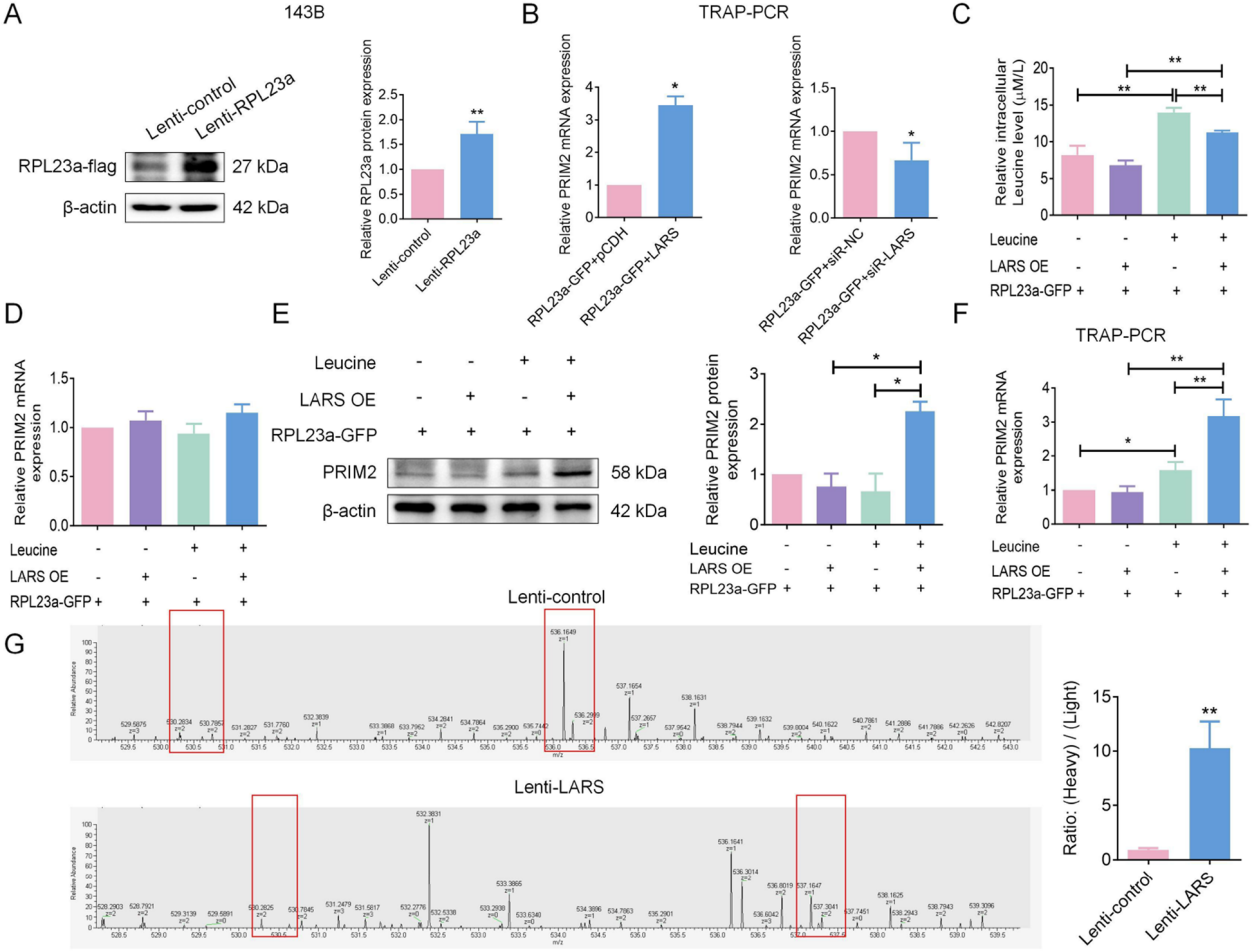
LARS enhances PRIM2 translation through leucine consumption. **A** WB analysis was used to detect the expression of RPL23a-flag in 143B cells transfected with either Lenti-RPL23a or Lenti-control vectors (*n* = 3; **p < 0.01). **B** TRAP-PCR was used to detect the abundance of PRIM2 mRNA in ribosome-bound mRNA complexes of RPL23a-overexpressing 143B cells transfected with LARS overexpression plasmid or siR-LARS (*n* = 3; *p < 0.05). **C** A leucine detection kit was used to measure the level of intracellular leucine in RPL23a stable overexpressing 143B cells transfected with LARS overexpression or control plasmids. Cells were incubated in the leucine-deficient medium for 24 h (*n* = 3; **p < 0.01). **D** RT-qPCR assays were used to detect the mRNA expression level of PRIM2 in RPL23a stable overexpressing 143B cells transfected with LARS overexpression or control plasmids. Cells were incubated in the leucine-deficient medium for 24 h (*n* = 3). (E) WB assays were used to detect the expression level of PRIM2 in RPL23a stable overexpressing 143B cells transfected with LARS overexpression or control plasmids (*n* = 3; *p < 0.05). **F** TRAP-PCR was used to detect the abundance of PRIM2 mRNA in the ribosome-bound mRNA fraction of RPL23a stable overexpressing 143B cells transfected with LARS overexpression or control plasmids. Cells were incubated in leucine-deficient medium for 24 h (*n* = 3; *p < 0.05, **p < 0.01). **G** The translational mechanism of LARS-mediated PRIM2 upregulation was investigated using SILAC quantification (*n* = 3; **p < 0.01)

Considering the essential role of RPL23a in the assembly of the 60S ribosome subunit, and its critical function in cellular protein synthesis [41], we constructed 143B cell line with RPL23a overexpression and verified the overexpression efficiency by WB for subsequent experiments (Fig. 13A). In 143B cells transfected to overexpress LARS, GFP-labeled RPL23a was utilized in a TRAP assay, and subsequently PCR analysis subsequent demonstrated an increased abundance of PRIM2 mRNA within the ribosome-fusion protein-mRNA complex. In contrast, PRIM2 mRNA abundance was correspondingly reduced in 143B cells transfected with LARS knockdown (Fig. 13B), suggesting that the expression of LARS positively regulated the translation efficiency of PRIM2. To investigate whether the LARS-medicated regulation of PRIM2 translation efficiency is leucine-dependent in OS, we initially measured the levels of free leucine in 143B cells, showing that cells transfected for LARS overexpression and labeled with RPL23a-GFP exhibited lower leucine levels than non-transfected overexpressing cells in the regular medium (Fig. 13C). Interestingly, PRIM2 mRNA levels in LARS-overexpressing cells with RPL23a-GFP-labeled showed no significant change with leucine-deficient or regular medium (Fig. 13D). However, PRIM2 protein levels were significantly upregulated in these cells with conventional cultures compared to leucine-deficient medium or non-transfected overexpressing cells (Fig. 13E). These findings suggest that LARS potentially employs leucine consumption to facilitate the translation of PRIM2 in OS cells, which was confirmed by subsequent TRAP-PCR detecting the translation efficiency of PRIM2 in these OS cells (Fig. 13F). Consistently, quantitative SILAC proteomics revealed that LARS overexpression significantly enhanced de novo PRIM2 synthesis. In SW1353 cells labeled with heavy L-leucine-^13^C₆, transduction with Lenti-LARS increased the synthesis rate of PRIM2 by 11.14-fold compared to Lenti-control-transduced cells (Fig. 13G), confirming that LARS upregulates PRIM2 protein abundance through leucine-sensitive translational potentiation.

**Fig. 14:**
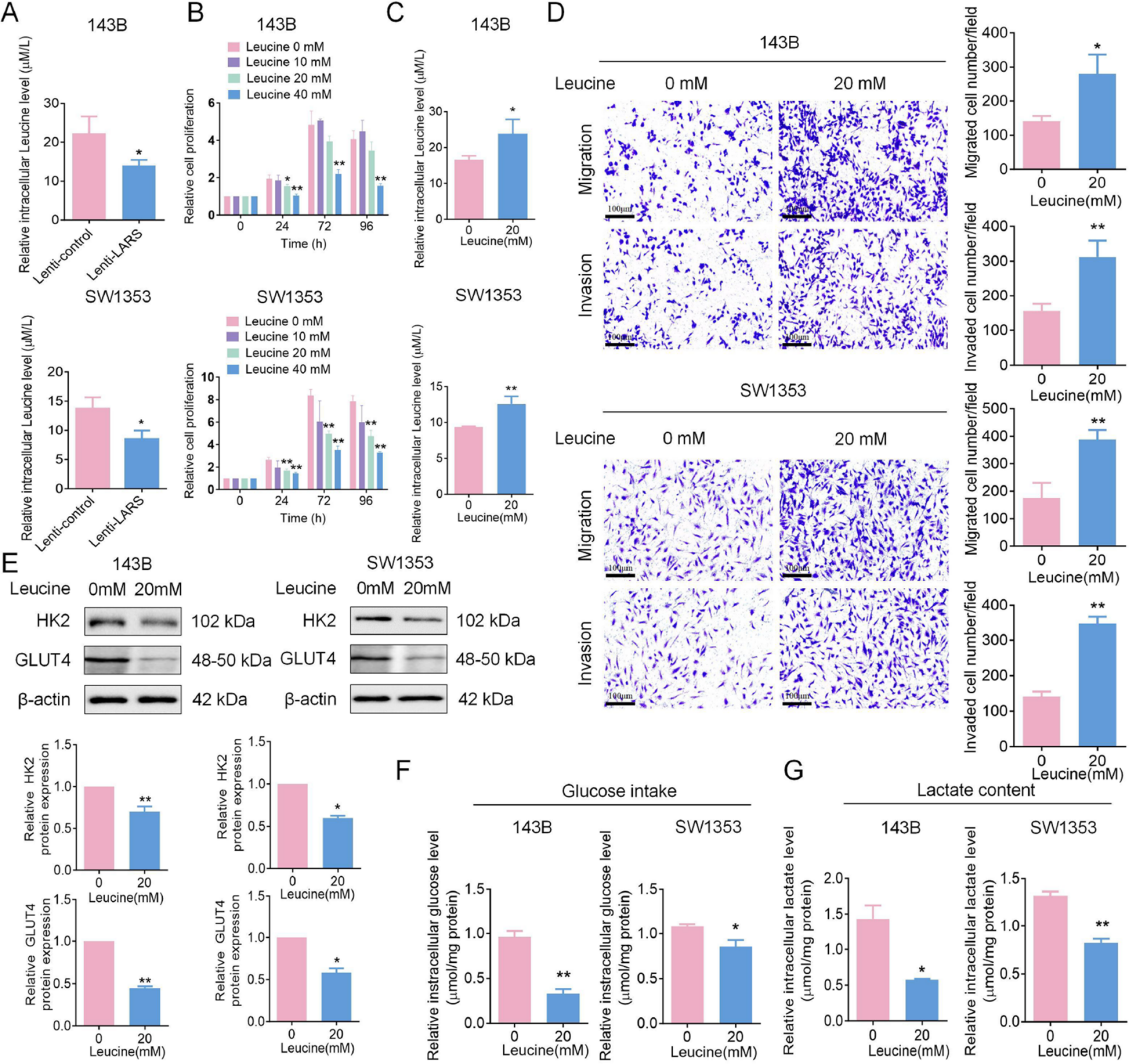
Exogenous leucine promotes proliferation and motility but inhibits glycolysis in OS cells. **A** Intracellular leucine level was measured in 143B and SW1353 cells transfected with Lenti-LARS or Lenti-control (*n* = 3; *p < 0.05). **B** MTT assays were used to detect the effect of various concentrations of leucine treatment for 24 h on the proliferation of 143B and SW1353 cells (*n* = 3; *p < 0.05, **p < 0.01). **C** Intracellular leucine level was measured in 143B and SW1353 cells after treatment with leucine (20 mM) for 24 h (*n* = 3; *p < 0.05, **p < 0.01). **D** Transwell assays were used to investigate the effect of leucine treatment (20 mM) for 24 h on the migration and invasion of 143B and SW1353 cells (scale bar: 200 μm; *n* = 3; *p < 0.05, **p < 0.01). **E** WB assays were used to detect the effect of leucine treatment (20 mM) for 24 h on the expression levels of HK2 and GLUT4 in 143B and SW1353 cells (*n* = 3; *p < 0.05, **p < 0.01). **F** and **G** The effect of LARS overexpression on glucose intake (**F**) and lactate content (**G**) in 143B and SW1353 cells treated with leucine (20 mM) was measured using glucose and lactate assay kits (*n* = 3; *p < 0.05, **p < 0.01)

Subsequently, we observed notable reductions in the leucine content within the stable overexpressed LARS OS cell lines (143B and SW1353) (Fig. 14A), confirming that elevated LARS expression leads to a consumption of leucine levels in OS cells. To assess the impact of LARS-modulated leucine, MTT assays were performed on OS cells exposed to various leucine doses over different durations. The results showed a dose-dependent decrease in cell proliferation at fixed time points, with the significant effects occurring at a concentration of 20 mM leucine following a 24-h treatment in both cell lines. Prolonged treatment increased cell proliferation with the same leucine doses (Fig. 14B), suggesting that leucine is a positive modulator of cell growth in these OS cells. To assess the acute effects of leucine concentration, we treated OS cells with 20 mM leucine for 24 h. Following treatment, leucine content significantly increased (Fig. 14C), confirming efficient uptake and retention by cells, paving the way for further analysis. Our data showed that leucine treatment enhanced cell migration and invasion in both 143B and SW1353 cells (Fig. 14D), suggesting that leucine promotes these phenotypic behaviors. Considering the established connection between amino acid metabolism and glycolysis, we proceeded to assess the expression levels of proteins associated with glycolysis in leucine-treated OS cells. Contrary to our expectations, the findings revealed a significant downregulation of key glycolytic markers, including HK2 and GLUT4, in both cell lines (Fig. 14E). Furthermore, there was a notable reduction in glucose uptake and lactate production in these cells compared to untreated controls (Fig. 14F-G), indicating that exogenous leucine suppresses glycolysis in OS cells. These results suggest that LARS regulates OS cell proliferation, motility, and glycolysis by consuming leucine, at least in part.

## Discussion

Although previous studies have indicated that LARS promotes proliferation in various cancers, the mechanisms underlying this process in OS cells remain elusive. In the present study, our findings indicate that LARS promotes OS proliferation by facilitating glycolysis (a hallmark of metabolic reprogramming in cancer) and mitigating ER stress on one hand, while PRIM2 mRNA translation is also mediated by LARS. Additionally, LARS mediates the translation of PRIM2 mRNA, which accelerates DNA replication, ultimately leading to the proliferation of OS cells. As a leucine sensor, LARS activates mTORC1 signaling and links amino acid availability to metabolic regulation [42]. LARS deficiency has also been shown to induce autophagy and metabolic stress *in vivo* [43]. In line with these findings, our data show that LARS overexpression enhances glycolysis, reduces ROS levels, and suppresses the PERK-ATF4-CHOP axis, supporting its role in metabolic reprogramming in OS. These findings not only elucidate the multifaceted role of LARS in metabolic reprogramming but also highlight the LARS/PRIM2 axis and the PRIM2-associated translation program as promising therapeutic targets for OS, offering new strategies to disrupt the metabolic machinery of osteosarcoma cells.

LARS promotes OS cell proliferation while suppressing migration and invasion, a pattern consistent with the migration-proliferation dichotomy (MPD) observed in various cancers. Studies in glioma and epithelial tumors have shown that proliferative and migratory phenotypes are often mutually exclusive, reflecting a trade-off between growth and motility [44–47]. In our study, LARS may shift OS cells toward a proliferative state by enhancing metabolic activity and activating the PERK-ATF4 pathway, potentially at the expense of cytoskeletal flexibility and invasion. These findings support that LARS regulates OS progression through phenotype selection under metabolic constraints.

Our findings position PRIM2 as a pivotal downstream effector through which LARS orchestrates ER stress mitigation, DNA replication, and proliferation in OS. This aligns with the established oncogenic role of PRIM2 across diverse cancers. Yin et al*.* found that PRIM2 promoted the proliferation and metastasis of pancreatic ductal adenocarcinoma [48]. Sun et al*.* highlighted PRIM2 as a marker of cell cycle progression and hyper-proliferation in glioma patients [49]. Similarly, Wang et al. showed that PRIM2 expression activated the cell cycle and promoted lung cancer progression [38]. While the function of PRIM2 in OS was previously unclear, our work not only confirms its similar pro-tumorigenic effect but also delineates a unique upstream regulatory mechanism, whereby its expression is translationally potentiated by LARS independently of mTOR signaling.

The present study reveals a leucine-centered regulatory mechanism that underpins the dual role of LARS in OS progression. We provide direct evidence that LARS, by consuming intracellular leucine, enhances the translation efficiency of PRIM2, thereby fueling DNA replication and cell proliferation while concurrently suppressing invasive motility. This finding resolves the apparent paradox between LARS-mediated invasion suppression and exogenous leucine-promoted invasion. We attribute this discrepancy to distinct leucine bioavailability dynamics: LARS overexpression creates a state of functional leucine depletion that prioritizes anabolic processes like proliferation, whereas leucine supplementation provides a surplus that supports both growth and motility programs. Thus, leucine itself acts as a metabolic rheostat, with its intracellular concentration fine-tuning the balance between proliferative and invasive states in OS. This concept is supported by prior studies showing that leucine metabolism is intricately linked to both protein synthesis [47] and glycolytic flux [50, 51], positioning our findings within a growing recognition of metabolic checkpoints in cancer cell decision-making.

The oncogenic role of LARS in OS unveils dual clinical implications: as a predictive biomarker and a therapeutic target. Clinically, elevated LARS expression correlates with enhanced glycolysis and DNA replication, which may predict sensitivity to cell cycle-dependent chemotherapeutics (e.g., methotrexate) while inversely associating with invasive phenotypes. Mechanistically, the LARS/PRIM2 axis represents a viable therapeutic target. Small-molecule inhibitors or siRNA-mediated silencing of LARS may disrupt translational efficiency and intensify endoplasmic reticulum stress in OS cells, thereby enhancing chemosensitivity. For translational implementation, combining LARS-targeted therapies with neoadjuvant chemotherapy represents a promising strategy. Previous studies have shown that targeting the tRNA synthetase therapeutic pathway may synergize with neoadjuvant chemotherapy to reduce tumor burden and mitigate metastasis [52, 53]. Future clinical trials may consider prioritizing the evaluation of LARS as a stratification marker for neoadjuvant chemotherapy response and investigating LARS-targeted agents in combination regimens. Additionally, exploring LARS-mediated metabolic reprogramming could uncover novel adjuvant strategies to overcome chemotherapy resistance in OS.

Although this study provides novel insights into the role of the LARS/PRIM2 axis in OS progression, several limitations should be acknowledged. First, while this study utilized established OS cell lines and xenograft models, we acknowledge that these systems may not fully recapitulate the genetic heterogeneity and complex TME of clinical OS. This inherent limitation is particularly relevant when interpreting translational findings related to patient samples. For instance, in our analysis of LARS expression (Fig. 1), the publicly available GEO datasets (GSE152048 and GSE270231) are constrained by the rarity of metastatic OS samples. The pulmonary metastatic foci and primary tumor samples were derived from different patients, and the cohort size remains limited despite our efforts to aggregate data. We have explicitly noted this context to ensure appropriate interpretation of the comparative results. Second, an intriguing observation from this study is that leucine supplementation enhanced cell migration and invasion while concurrently downregulating key glycolytic markers HK2 and GLUT4. We propose that restored intracellular leucine levels, compensating for LARS-induced depletion, may activate other pathways to meet the biosynthetic and energetic demands of cell motility. Notably, LARS overexpression may also regulate the metastatic process by influencing the seeding efficiency of OS cells to the lung microenvironment, thereby affecting their subsequent colonization and outgrowth into metastatic nodules. Future studies could further explore the underlying mechanisms by which LARS modulates OS cell adhesion, extravasation, and adaptation to the lung microenvironment, which would provide deeper insights into the role of the LARS/PRIM2 axis in OS metastasis. Third, a limitation of this study is that comprehensive clinical variables, including treatment response, metastatic sites, and therapy regimens, were not uniformly available across the public datasets analyzed, which is an inherent constraint of retrospective survival analyses based on integrated databases. Mechanistically, while we identified LARS/PRIM2's role in glycolysis, DNA replication, and ER stress, other critical pathways that may crosstalk with this axis remain unexplored. Moreover, the exact interplay between LARS/PRIM2 signaling, ER stress modulation, and glycolytic regulation remains to be clarified. Finally, the translational potential of targeting LARS faces challenges, including potential on-target toxicity due to LARS's essential role in protein synthesis and the lack of combination therapy studies with conventional chemotherapeutics or immunotherapy. Future work may validate these findings in patient-derived models or primary cells and evaluate tumor-selective LARS inhibition strategies.

## Conclusion

This study demonstrates that LARS expression is significantly upregulated in OS, where its overexpression promotes tumor cell proliferation while suppressing invasion and migration. Conversely, LARS silencing induces cell cycle arrest. Mechanistically, LARS upregulation drives metabolic reprogramming through enhanced glycolysis and DNA replication, coupled with reduced ER stress. Furthermore, LARS transcriptionally upregulates the oncogenic factor PRIM2, with leucine-dependent potentiation of PRIM2 translational efficiency. Overall, these findings underscore the critical role of the LARS/PRIM2 axis in driving OS tumorigenesis, mainly through the mitigation of endoplasmic reticulum stress and activation of translation processes, suggesting potential therapeutic targets for OS treatment.

## Supplementary Information

Supplementary Material 1: Figure S1. Effects of LARS overexpression on 143B cells. (A) WB confirmation of LARS overexpression in 143B cells transfected with the LARS plasmid (LARS) vs. an empty vector control (pCDH) (*n*=3; *p<0.05). (B) Transwell migration and invasion assays of 143B cells upon LARS overexpression (scale bar: 100 μm; *n*=3; **p<0.01). (C) MTT proliferation assay of 143B cells upon LARS overexpression (*n*=3; **p<0.01). (D) Colony formation assay of 143B cells upon LARS overexpression (*n*=3; **p< 0.01). (E) Cell cycle analysis by flow cytometry in 143B cells upon LARS overexpression, and the cell cycle distribution was evaluated immediately 24 h after transduction (*n*=3; *p<0.05). (F) EdU (red) and DAPI (blue) staining to assess proliferation in 143B cells upon LARS overexpression (magnification: 630×; scale bar: 20 μm; *n*=3; **p<0.01). Figure S2. Silencing of LARS suppresses the malignant progression of MG-63 cells. (A) WB analysis was used to detect knockdown efficiency at three LARS loci in MG-63 cells (*n*=3; *p<0.05). (B) Transwell assays were used to examine the effect of LARS silencing on the migration and invasion of MG-63 cells (scale bar: 100 μm; *n*=3; *p<0.05,**p<0.01). (C) MTT assays were used to detect the effect of LARS silencing on the proliferation of MG-63 cells (*n*=3; *p<0.05). (D) Colony formation assays were employed to investigate the effect of LARS silencing on the colony formation of MG-63 cells (*n*=3; **p<0.01). (E) Flow cytometry was used to detect the effect of LARS silencing on the cell cycle of MG-63 cells at 48 h post-transfection (*n*=3; *p<0.05). (F) EdU (red) and DAPI (blue) staining was used to assess the effect of LARS silencing on the proliferation of MG-63 cells (magnification: 630×; scale bar: 20 μm;*n*=3; *p<0.05). Figure S3. Silencing of LARS suppresses the malignant progression of HOS cells. (A) WB analysis of knockdown efficiency at three LARS-targeting loci in HOS cells (*n*=3; *p<0.05,**p<0.01). (B) Transwell migration and invasion assays of HOS cells upon LARS knockdown (scale bar: 100 μm;*n*=3; *p<0.05, **p<0.01). (C) MTT proliferation assay of HOS cells upon LARS knockdown (*n*=3; *p<0.05). (D) Colony formation assay of HOS cells upon LARS knockdown (*n*=3; *p<0.05). (E) Cell cycle analysis by flow cytometry in HOS cells following LARS knockdown, with cell cycle distribution evaluated immediately at 48 h after transfection (*n*=3; *p<0.05,**p<0.01). (F) EdU (red) and DAPI (blue) staining to assess proliferation in HOS cells upon LARS knockdown (magnification: 630×; scale bar: 20 μm;*n*=3; *p<0.05). Figure S4. LARS overexpression induces DNA replication and modulates ER stress in SW1353 cells. (A) MTT assay assessing the viability of SW1353 cells after treatment with different concentrations of CCT020312 for 24 h (*n*=3; *p<0.05, **p<0.01). (B) MTT assay evaluating the effect of LARS overexpression combined with CCT020312 (1 μM) treatment on the proliferation of SW1353 cells (*n*=3; *p<0.05,**p<0.01). Flow cytometry analysis of ROS levels (C) and measurement of ER calcium ion concentration (D) in SW1353 cells upon LARS stable overexpression combined with CCT020312 treatment (*n*=3; **p<0.01). (E) WB analysis of the ER stress pathway (p-PERK, PERK, ATF4, CHOP) in SW1353 cells upon LARS stable overexpression combined with CCT020312 treatment (*n*=3; **p<0.01). (F) Flow cytometric detection of ROS levels in SW1353 cells upon LARS knockdown combined with NAC treatment (*n*=3; **p<0.01). (G) WB analysis of the ER stress pathway (p-PERK, PERK, ATF4, CHOP) in SW1353 cells upon LARS knockdown combined with NAC treatment (*n*=3; **p<0.01). Figure S5. LARS overexpression induces DNA replication and ER stress in 143B cells. (A) MTT assays were used to assess the cell viability of 143B cells after treatment with different concentrations of CCT020312 for 24 h (*n*=3; *p<0.05, **p<0.01). (B) MTT assays were used to assess the effect of LARS overexpression combined with CCT020312 treatment (2 μM) on the proliferation of 143B cells (*n*=3; *p<0.05,**p<0.01). Flow cytometry analysis of ROS levels (C) and measurement of ER calcium ion concentration (D) in SW1353 cells upon LARS stable overexpression combined with CCT020312 treatment (*n*=3; *p<0.05, **p<0.01). (E) WB assays were used to assess the effect of LARS stable overexpression combined with CCT020312 treatment on the expression levels of p-PERK, PERK, ATF4, and CHOP in 143B cells (*n*=3; *p<0.05, **p<0.01). (F) Flow cytometric detection of ROS levels in 143B cells upon LARS silencing combined with NAC treatment (*n*=3; *p<0.05, **p<0.01). (G) WB assay was used to detect the effect of LARS knockdown combined with NAC treatment on the expression levels of p-PERK, PERK, ATF4 and CHOP in 143B cells cells (*n*=3; **p<0.01). Figure S6. Validation of LARS manipulation and its effects in MG-63 and K7M2 OS cell models *in vivo*. (A) MG-63 cells were transfected with either Lenti-sh-LARS or Lenti-sh-control vectors, and the transfection efficiency was assessed using WB assays (*n*=3; **p<0.01); (B) Picture was taken after the tumor was removed. The volume and weight of the tumors were recorded (*n*=6; **p<0.01). (C) K7M2 cells were transfected with either Lenti-LARS or Lenti-control vectors. The transfection efficiency was assessed using WB assays (*n*=3; *p<0.05). Figure S7. Knockdown of PRIM2 reverses the LARS-mediated cell proliferation and DNA replication in SW1353 cells. (A) MTT assay assessing the effect of LARS stable overexpression combined with PRIM2 knockdown on the proliferation of SW1353 cells (*n*=3; *p<0.05). (B) Flow cytometry analysis of the cell cycle in SW1353 cells stably overexpressing LARS following PRIM2 knockdown, performed 24 h after PRIM2 transfection (*n*=3; *p<0.05, **p<0.01). (C) EdU (red) and DAPI (blue) staining to assess the effect of LARS stable overexpression combined with PRIM2 knockdown on the proliferation of SW1353 cells (Magnification: 630×; scale bar: 20 μm; *n*=3; *p<0.05, **p<0.01). (D) WB analysis of DNA replication and proliferation markers (Ki-67, PCNA, MCM2, ORC1) in SW1353 cells upon LARS stable overexpression combined with PRIM2 knockdown (*n*=3; *p<0.05, **p<0.01). Figure S8. Knockdown of PRIM2 reverses the LARS-mediated cell proliferation and DNA replication in 143B cells. (A) MTT assays were used to detect the effect of LARS stable overexpression combined with PRIM2 knockdown on the proliferation of 143B cells (*n*=3; **p<0.01). (B) Flow cytometry assays were used to detect the effect of stable LARS overexpression combined with PRIM2 knockdown on the cell cycle of 143B cells, performed 24 h after PRIM2 transfection (*n*=3; *p<0.05, **p<0.01). (C) EdU (red) and DAPI (blue) staining was used to assess the effect of LARS stable overexpression combined with PRIM2 knockdown on the proliferation of 143B cells (Magnified 630×; scale bar: 20 μm; *n*=3; **p<0.01). (D) WB assays were used to assess the effect of LARS stable overexpression combined with PRIM2 knockdown on the expression levels of Ki-67, PCNA, MCM2, and ORC1 in 143B cells (*n*=3; *p<0.05,**p<0.01). Figure S9. LARS modulates cell proliferation and DNA replication in OS cells by upregulating PRIM2 expression. (A) SW1353 cells were transfected with PRIM2 overexpression plasmids or empty plasmids (pCDH) for 24 h and 48 h (*n*=3; *p<0.05). (B) MTT assays were employed to evaluate the impact on the proliferative capacity of 143B cells 24 h after transfection with PRIM2 overexpression or siR-LARS, either separately or in combination (*n*=3; **p<0.01). (C) Flow cytometry assays were used to assess the impact on the cell cycle of 143B cells, performed immediately 24 h after siR-LARS transfection (*n*=3; scale bar: 20 μm; *p<0.05, **p<0.01). (D) EdU (red) and DAPI (blue) staining was employed to evaluate the impact on the proliferative capacity of 143B cells 24 h after transfection with PRIM2 overexpression or siR-LARS (Magnified 630×; scale bar: 20 μm; *n*=3; *p<0.05, **p<0.01). (E) WB assays were used to assess the impact on the expression of Ki-67, PCNA, MCM2, and ORC1 in 143B cells 24 h after transfection with PRIM2 overexpression or LARS siRNA (*n*=3; *p<0.05, **p<0.01).

Supplementary Material 2: Table S1. Clinicopathological characteristics of osteosarcoma samples used for IHC analysis. Table S2. Mean cell-type proportions in primary and metastatic OS samples.

## Data Availability

The datasets supporting the conclusions of this article are included within the article (and its additional files). The datasets used and/or analyzed during the current study are available from the corresponding author on reasonable request.

## Abbreviations

OS: Osteosarcoma
ATP: Adenosine Triphosphate
mTORC1: Mammalian Target of Rapamycin Complex 1
ANGPTL4: Angiopoietin-Like 4
ARS: Aminoacyl-tRNA Synthetase
LARS/LARS1: Leucyl-tRNA Synthetase
ER: Endoplasmic Reticulum
PRIM2: Primase P58 Subunit 2
RPL23a: Ribosomal Protein L23a
MTT: Methyl Thiazolyl Tetrazolium
ROS: Reactive Oxygen Species
NAC: N-Acetylcysteine
PERK: PKR-like Endoplasmic Reticulum Kinase
EdU: 5-Ethynyl-2'-deoxyuridine
IHC: Immunohistochemistry
DAB: 3,3'-Diaminobenzidine
WB: Western Blotting
RT-qPCR: Real-Time quantitative Polymerase Chain Reaction
cDNA: Complementary DNA
PBS: Phosphate-Buffered Saline
DMSO: Dimethyl Sulfoxide
OD: Optical Density
PI: Propidium Iodide
FBS: Fetal Bovine Serum
IOD: Integrated Optical Density
ROI: Region of Interest
PVDF: Polyvinylidene Difluoride
SDS-PAGE: Sodium Dodecyl Sulfate–Polyacrylamide Gel Electrophoresis
BCA: Bicinchoninic Acid
TCA: Trichloroacetic Acid
TEAB: Triethylammonium Bicarbonate Buffer
DTT: Dithiothreitol
IAA: Iodoacetamide
PMSF: Phenylmethanesulfonyl Fluoride
TRAP-PCR: Telomeric Repeat Amplification Protocol-PCR
CHX: Cycloheximide
SILAC: Stable Isotope Labeling by Amino Acids in Cell Culture
scRNA-seq: Single-Cell RNA Sequencing
GEO: Gene Expression Omnibus
PCA: Principal Component Analysis
UMAP: Uniform Manifold Approximation and Projection
SNN: Shared Nearest Neighbor
CNV: Copy Number Variation
DEG: Differentially Expressed Gene
GO: Gene Ontology
KEGG: Kyoto Encyclopedia of Genes and Genomes
TCGA: The Cancer Genome Atlas
DEP: Differentially Expressed Protein
CDS: Coding Sequence
siRNA: Small Interfering RNA
shRNA: Short Hairpin RNA
HK2: Hexokinase 2
GLUT4: Glucose Transporter 4
PCNA: Proliferating Cell Nuclear Antigen
MCM2: Minichromosome Maintenance Complex Component 2
ORC1: Origin Recognition Complex Subunit 1
IRE-1α: Inositol-Requiring Enzyme 1 Alpha
ATF6: Activating Transcription Factor 6
GRP78: Glucose-Regulated Protein 78
UPR: Unfolded Protein Response
p-PERK: Phosphorylated PERK
ATF4: Activating Transcription Factor 4
CHOP: C/EBP Homologous Protein
SPP1: Secreted Phosphoprotein 1
IBSP: Integrin-Binding Sialoprotein
BEX4: Brain Expressed X-Linked 4
PCP4: Purkinje Cell Protein 4
MPD: Migration-Proliferation Dichotomy
