# Supplementary figures and images for: LARS promotes osteosarcoma proliferation through leucine-dependent PRIM2 translation and DNA replication activation
Source: J Exp Clin Cancer Res. 2026 Mar 14;45:103. doi: 10.1186/s13046-026-03691-w (PMC13101216; doi:10.1186/s13046-026-03691-w)

Figure S1

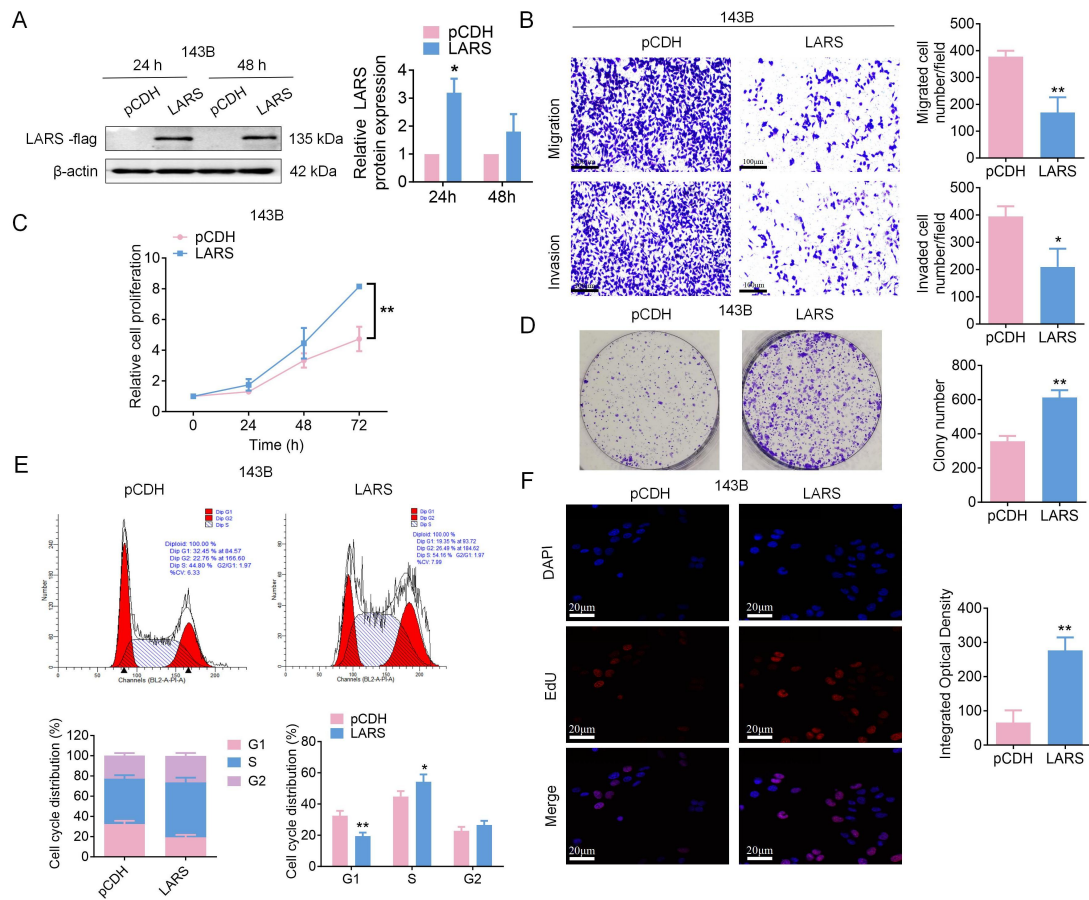

Figure S2

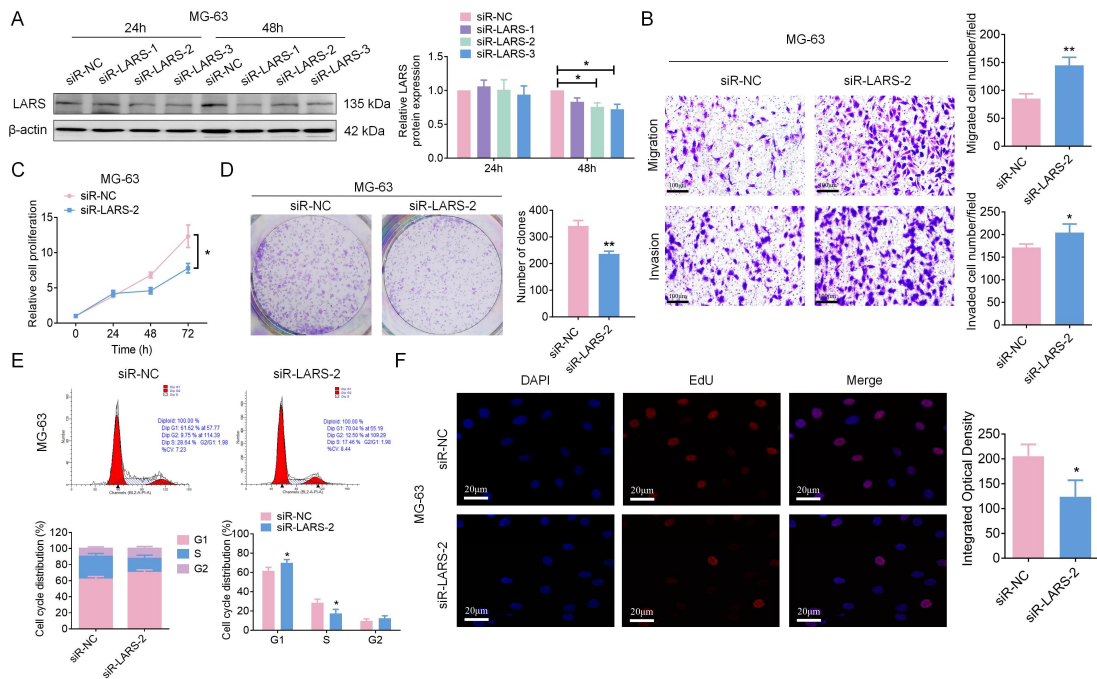

Figure S3

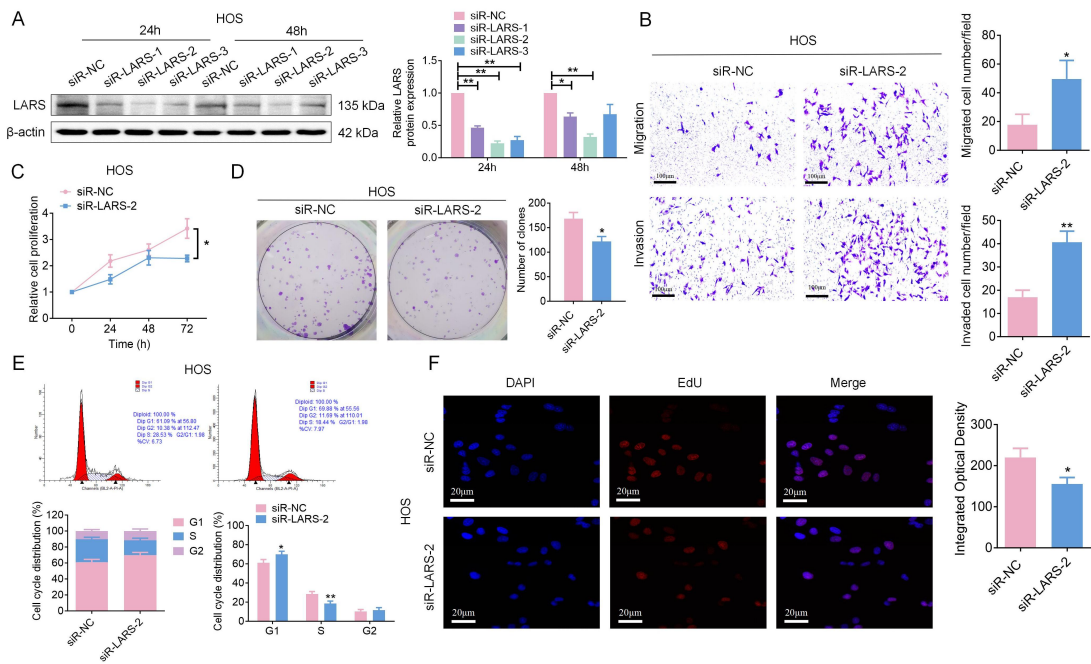

Figure S4

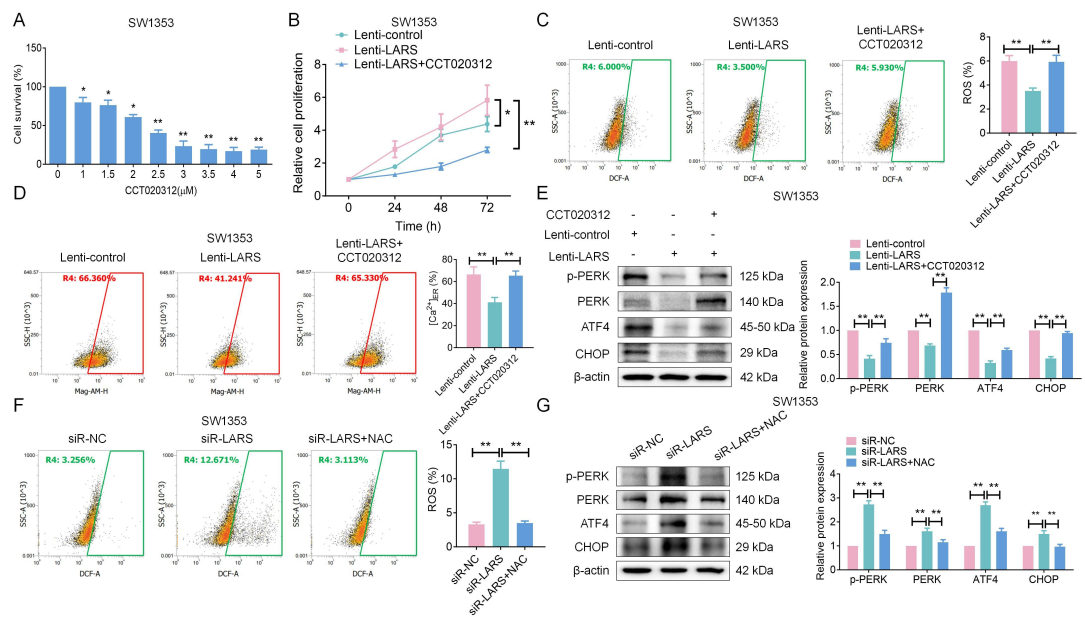

Figure S5

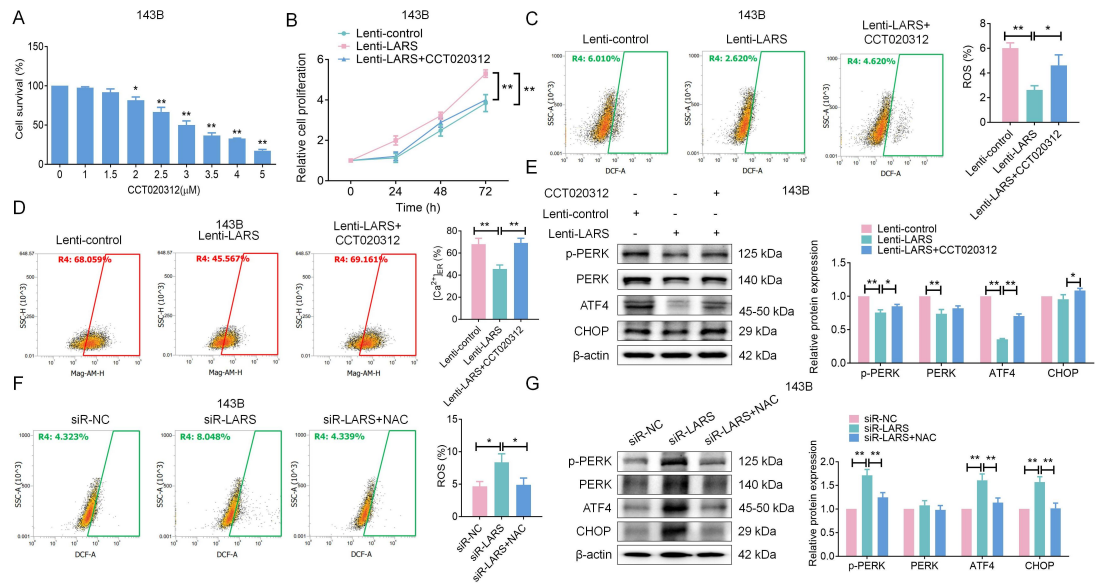

Figure S6

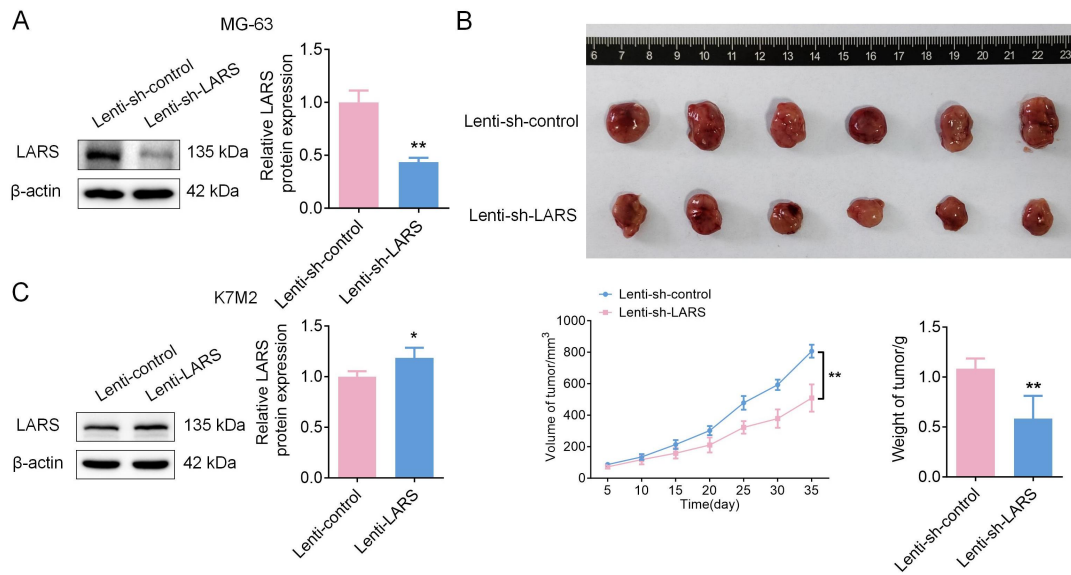

Figure S7

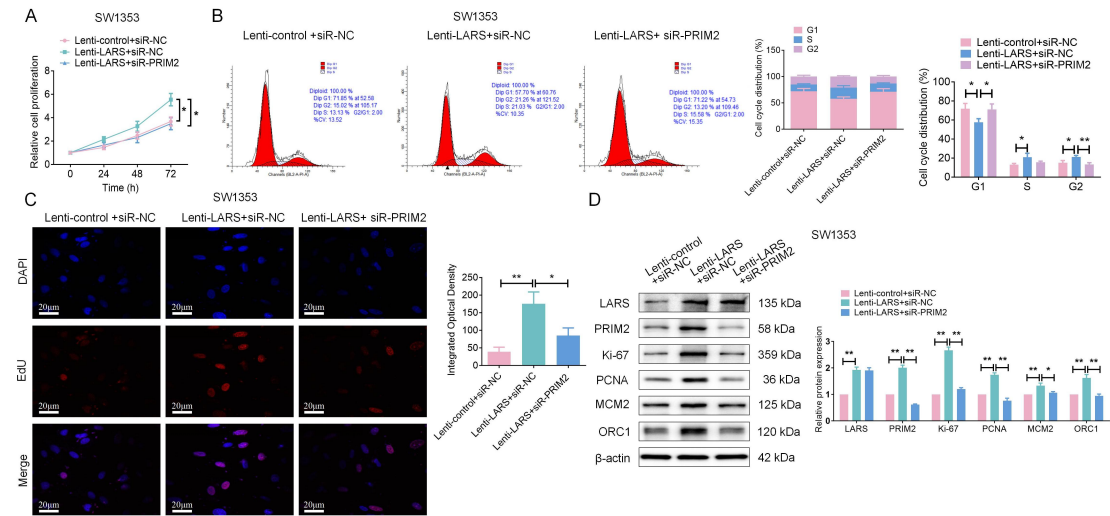

Figure S8

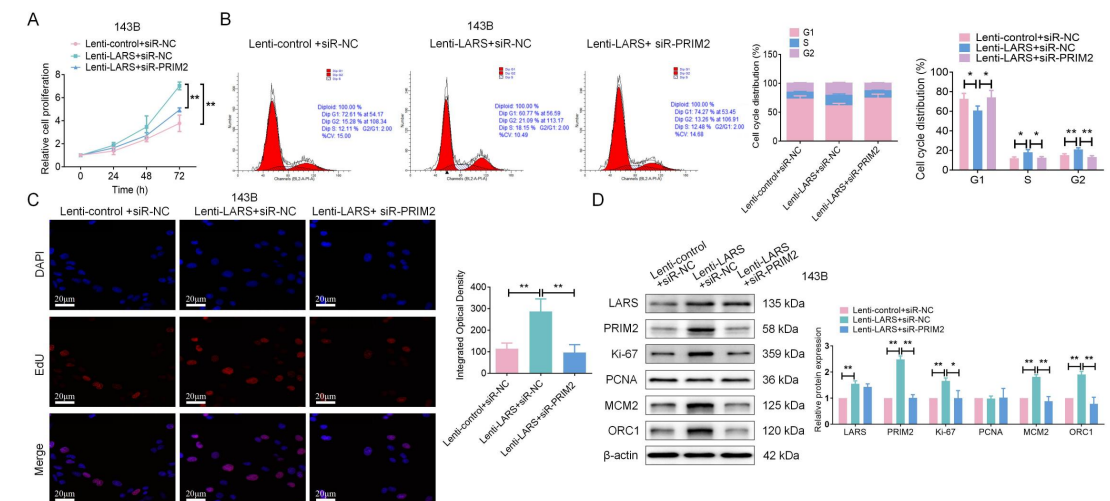

Figure S9

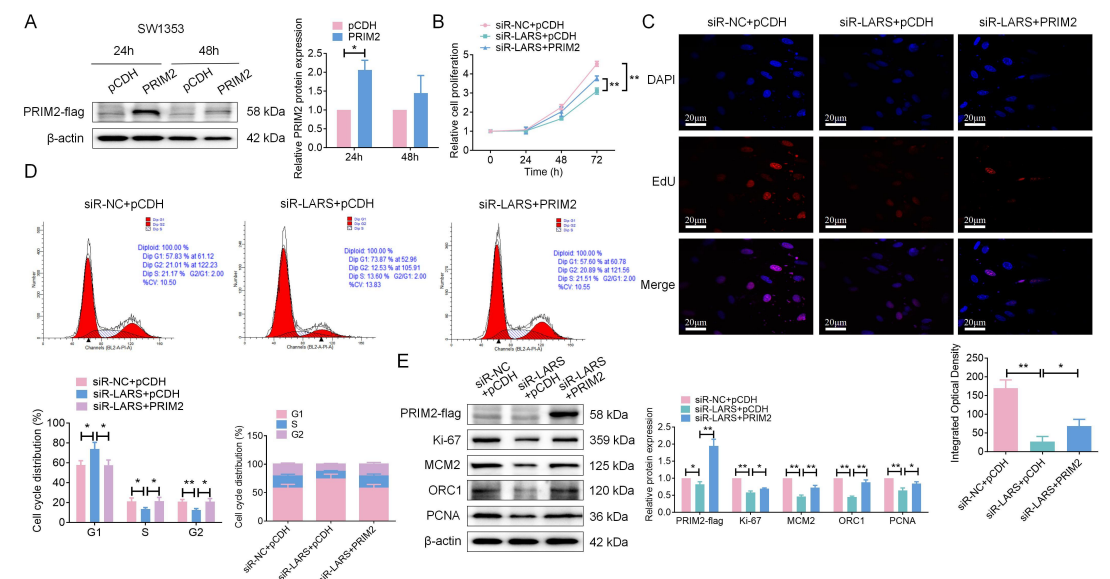

Supplement: Supplementary file 1 — Supplementary Material 1: Figure S1. Effects of LARS overexpression on 143B cells. (A) WB confirmation of LARS overexpression in 143B cells transfected with the LARS plasmid (LARS) vs. an empty vector control (pCDH) (n=3; *p<0.05). (B) Transwell migration and invasion assays of 143B cells upon LARS overexpression (scale bar: 100 μm; n=3; **p<0.01). (C) MTT proliferation assay of 143B cells upon LARS overexpression (n=3; **p<0.01). (D) Colony formation assay of 143B cells upon LARS overexpression (n=3; **p< 0.01). (E) Cell cycle analysis by flow cytometry in 143B cells upon LARS overexpression, and the cell cycle distribution was evaluated immediately 24 h after transduction (n=3; *p<0.05). (F) EdU (red) and DAPI (blue) staining to assess proliferation in 143B cells upon LARS overexpression (magnification: 630×; scale bar: 20 μm; n=3; **p<0.01). Figure S2. Silencing of LARS suppresses the malignant progression of MG-63 cells. (A) WB analysis was used to detect knockdown efficiency at three LARS loci in MG-63 cells (n=3; *p<0.05). (B) Transwell assays were used to examine the effect of LARS silencing on the migration and invasion of MG-63 cells (scale bar: 100 μm; n=3; *p<0.05,**p<0.01). (C) MTT assays were used to detect the effect of LARS silencing on the proliferation of MG-63 cells (n=3; *p<0.05). (D) Colony formation assays were employed to investigate the effect of LARS silencing on the colony formation of MG-63 cells (n=3; **p<0.01). (E) Flow cytometry was used to detect the effect of LARS silencing on the cell cycle of MG-63 cells at 48 h post-transfection (n=3; *p<0.05). (F) EdU (red) and DAPI (blue) staining was used to assess the effect of LARS silencing on the proliferation of MG-63 cells (magnification: 630×; scale bar: 20 μm;n=3; *p<0.05). Figure S3. Silencing of LARS suppresses the malignant progression of HOS cells. (A) WB analysis of knockdown efficiency at three LARS-targeting loci in HOS cells (n=3; *p<0.05,**p<0.01). (B) Transwell migration and inv [file 13046_2026_3691_MOESM1_ESM.pdf]
