# Supplementary material for: LARS promotes osteosarcoma proliferation through leucine-dependent PRIM2 translation and DNA replication activation
Source: J Exp Clin Cancer Res. 2026 Mar 14;45:103. doi: 10.1186/s13046-026-03691-w (PMC13101216; doi:10.1186/s13046-026-03691-w)
Supplement: Supplementary file 2 — Supplementary Material 2: Table S1. Clinicopathological characteristics of osteosarcoma samples used for IHC analysis. Table S2. Mean cell-type proportions in primary and metastatic OS samples. [file 13046_2026_3691_MOESM2_ESM.docx]

**Table S1. Clinicopathological characteristics of osteosarcoma samples used for IHC analysis.**

| **Patient ID** | **Age (years)** | **Sex** | **Clinical stage** | **Sample type** | **Metastatic status** | **Metastatic site** |
| --- | --- | --- | --- | --- | --- | --- |
| 1 | 18 | Male | IIB | Primary tumor & Adjacent tissue | Primary tumor (no metastasis) | N/A |
| 2 | 14 | Male | III | Primary tumor & Adjacent tissue & Metastatic tissue | Primary tumor with synchronous metastasis | Lung |
| 3 | 14 | Male | III | Primary tumor & Adjacent tissue & Metastatic tissue | Primary tumor with synchronous metastasis | Lung |
| 4 | 15 | Female | III | Primary tumor & Adjacent tissue & Metastatic tissue | Primary tumor with synchronous metastasis | Lung |
| 5 | 11 | Male | III | Primary tumor & Adjacent tissue & Metastatic tissue | Primary tumor with synchronous metastasis | Lung |
| 6 | 13 | Female | III | Primary tumor & Adjacent tissue & Metastatic tissue | Primary tumor with synchronous metastasis | Lung |
| 7 | 11 | Female | III | Primary tumor & Adjacent tissue & Metastatic tissue | Primary tumor with synchronous metastasis | Lung |
| 8 | 8 | Male | IIB | Primary tumor & Adjacent tissue | Primary tumor (no metastasis) | N/A |

**Table S2. Mean cell-type proportions in primary and metastatic OS samples.**

| **Cell type** | **Primary OS** | **Metastatic OS** |
| --- | --- | --- |
| B Cells | 0.66% | 2.44% |
| Basophils | / | 1.58% |
| Endothelial Cells | 5.36% | 2.90% |
| Epithelial Cells | / | 2.64% |
| Fibroblasts | / | 1.66% |
| MSCs | 2.35% | 1.47% |
| Macrophages | 28.50% | 6.83% |
| Malignant osteoblasts | 17.34% | 13.07% |
| Myeloid cells | 2.13% | 0.37% |
| NK Cells | / | 2.89% |
| Osteoblastic OS | 23.80% | 40.22% |
| Osteoclastic OS | 11.67% | 5.90% |
| Pericyte | 3.99% | 1.28% |
| T Cells | 4.20% | 16.75% |
